# Development of a Digital Twin Monitoring Framework for Blade Polishing Robot Process Based on Cloud–Edge–Device

**DOI:** 10.3390/s26165150

**Published:** 2026-08-14

**Authors:** Nina Wang, Guohui Zhang, Yantao Ma, Jiahao Gao, Lijuan Ren, Guangpeng Zhang

**Affiliations:** School of Mechanical and Precision Instrument Engineering, Xi’an University of Technology, Xi’an 710048, China; 18792511708@163.com (N.W.); zgh18303588542@163.com (G.Z.); 15686080848@163.com (Y.M.); gjh00024@163.com (J.G.); rljuan@xaut.edu.cn (L.R.)

**Keywords:** abrasive belt grinding, digital twin monitoring framework, cloud–edge–device architecture, grinding robot, real-time monitoring

## Abstract

Digital twins are digital representations of physical entities that enable real-time updates through data transmission between the physical and virtual domains. Based on a cloud–edge–device framework, this paper investigates methods for real-time data transmission, processing, and storage during the polishing process of a belt grinding robot. On this basis, a digital twin monitoring framework is constructed for blade-specific belt grinding robots. First, a virtual robot model was constructed using a joint modeling workflow in SolidWorks 2025 and 3ds Max 2025, incorporating a lightweight high-fidelity mesh processing algorithm based on the QEM method. Second, a data acquisition and transmission architecture was proposed for the belt grinding robot, enabling data reading, writing, and real-time monitoring during machining, as well as establishing a cloud–edge–device database. Finally, a cloud–edge–device digital twin monitoring framework for the blade belt grinding robot was developed, based on real-time monitoring of grinding process data. This work establishes a foundational data acquisition and visualization platform for the blade grinding robot, providing the necessary cyber-physical infrastructure, which future predictive models can develop and validate.

## 1. Introduction

Blades are critical components of power equipment, including aeroengines, steam turbines, and gas turbines [1] (p. 34). Belt grinding, as the final step in blade finishing, directly determines the final machining quality and service performance of the blade [2] (p. 34). Due to their high flexibility and adaptability, grinding robots have become the primary equipment for blade grinding operations and are widely adopted in industrial applications [3] (p. 34). However, owing to the elasticity of the abrasive belt and contact wheel, combined with the relatively low rigidity of the robot structure, the actual grinding depth deviates significantly from the theoretical value, making it difficult to achieve the required machining accuracy for ground blades [4] (p. 34). Recently, advanced finishing processes such as cryogenic-assisted polishing have been explored for difficult-to-machine aerospace materials including additively manufactured Inconel 718. Pérez et al. [5] (p. 34) employed a radial abrasive brush combined with cryogenic cooling for polishing additively manufactured Inconel 718, providing new insights into surface quality optimization for high-precision aeroengine components. Therefore, it is necessary to accurately monitor key process parameters such as material removal rate, tool wear state, and grinding pressure during the actual grinding process, so that grinding parameters can be adjusted and grinding allowances can be planned in real time [6] (p. 34).

Digital twin technology is an important technical means to realize the digital monitoring process of manufacturing equipment and has been widely used in the field of digital manufacturing. However, belt grinding is different from other rigid machining processes. The grinding process involves massive amounts of multi-modal data, including the robot’s own joint information, cutting process data related to the actual material removal rate such as sparks, sounds, and vibrations generated during grinding, and belt grinding parameters (belt speed, belt width), making it difficult to realize a digital twin in the robot blade grinding process. Therefore, how to realize digital monitoring in the blade grinding process and apply it to actual grinding to solve the problem of insufficient precision in robot blade grinding has become a hot research topic for scholars at home and abroad.

Considerable research has been conducted on condition monitoring of industrial robots. Garcia et al. [7] (p. 34) developed a system-integration-centered robot for human–robot collaborative grinding. Souma et al. [8] (p. 34) established a multi-sensor system for online robotic arm health monitoring using wavelet analysis and decision trees combined with machine learning for anomaly detection. Yang et al. [9] (p. 34) constructed a cloud-manufacturing-based monitoring platform leveraging 5G networks, covering message delivery, data storage, visualization, and AI model training. Ayankoso et al. [10] (p. 34) proposed a kinematics- and dynamics-based digital twin scheme employing deep learning for fault prediction. Alobaidy et al. [11] (p. 34) employed an ADXL345 accelerometer to capture vibration signals from a robotic arm, and applied discrete and slantlet wavelet transformations for real-time fault diagnosis of industrial robots. Zhou et al. [12] (p. 34) developed a B/S architecture-based remote monitoring platform using an embedded controller for real-time data collection. Li et al. [13] (p. 35) integrated onboard multi-sensors to continuously monitor operational parameters including position, velocity, and power. These studies provide a solid foundation for robot monitoring, yet their efficiency in grinding data analysis remains limited. Although the aforementioned studies have provided a relatively comprehensive overview of industrial robot condition monitoring, few of them have directly addressed the application of digital twin technology to the robotic grinding/polishing domain. Recently, Zhang et al. [14] (p. 35) developed an industrial robot polishing system based on a digital twin for titanium alloy brake shells, enabling real-time process monitoring and parameter optimization. Moreover, a recent digital-twin-driven simulation method [15] (p. 35) incorporating dynamic trajectory tracking errors has achieved real-time material removal simulation with significantly improved efficiency.

In summary, numerous domestic and international studies have made substantial progress in the condition monitoring of industrial robots. Nevertheless, with the continuous emergence of advanced software frameworks, innovative communication protocols, and high-efficiency data storage technologies, traditional processing methods still suffer from low efficiency in grinding data analysis. In this context, cloud computing, with its powerful computational capability, provides an effective solution to such limitations. To further improve system service quality [16] (p. 35), edge computing has been introduced as a promising distributed computing paradigm. Unlike cloud computing, edge computing establishes a buffer layer between physical devices and cloud servers, enabling localized lightweight computation, real-time control, and data storage at the edge side [17] (p. 35). Recently, computer vision techniques have been introduced into the finishing processes of aeroengine components. González et al. [18] (p. 35) proposed an adaptive edge finishing method based on robot-assisted computer vision, which processes detected contour deviation signals to rapidly identify edges and burrs while dynamically adjusting toolpaths.

Ma et al. [19] (p. 35) proposed an integrated sustainable benchmark architecture based on cloud–edge collaboration and big data analysis. Taking energy consumption analysis as the workshop evaluation benchmark, this architecture can monitor and analyze energy utilization patterns, thereby facilitating the long-term goal of sustainable development in energy-intensive manufacturing industries. Guo et al. [20] (p. 35) proposed a deeply integrated human–machine–object manufacturing system based on cloud–edge collaboration, which is defined as a cloud–edge–end collaborative manufacturing system. This system realizes the in-depth integration of manufacturing resources, computer systems, and multi-dimensional manufacturing elements including personnel, equipment, and physical objects. Zhang et al. [21] (p. 35) presented an integrated performance-driven process monitoring framework, which deploys diverse monitoring tasks at the device, edge, and cloud layers respectively. Xie et al. [22] (p. 35) proposed an Internet of Things (IoT)-based intelligent manufacturing method. By introducing blockchain technology and cloud–edge collaborative computing, this method further improves system response efficiency and computing resource utilization. In terms of scalable data processing, Tapia et al. [23] (p. 35) constructed a data processing pipeline for aeronautical machine tool monitoring using Apache NiFi, Kafka, and Spark Streaming, demonstrating the effectiveness of open-source big data tools in manufacturing monitoring scenarios. These studies validate the advantages of cloud–edge–end architecture in terms of low latency and high practicality; however, its application in blade belt grinding robots remains limited. In addition to the aforementioned studies on robot condition monitoring and cloud–edge–end architectures, recent advances in polishing and deburring processes for aeroengine components are also highly relevant to the present work. For instance. Rodríguez et al. [24] (p. 35) developed an automated edge finishing cell for large turbine casings using both defined multi-edge and abrasive tools, demonstrating the feasibility of robotic systems for high-value aerospace parts. González et al. [25] (p. 35) further investigated the surface roughness evolution when brushing heat-resistant alloy components, providing valuable insights into the finishing quality of difficult-to-machine materials. These studies highlight the growing importance of integrating process monitoring with advanced finishing techniques, which motivates our development of a digital twin framework for the blade polishing robot. In conclusion, owing to its low latency and high practicability, the cloud–edge–end collaborative architecture is highly suitable for various industrial application scenarios. However, this advanced architecture has rarely been applied to the field of blade belt grinding robots.

On the other hand, the data involved in grinding operations covers not only static information such as equipment parameters, workpiece attributes, material properties, and process specifications but also real-time operational data including grinding force, temperature, surface roughness, surface integrity, spark images, and acoustic signals. Furthermore, the speed of data acquisition and transmission is a critical factor that determines the synchronization performance of digital twin systems. Kene et al. [26] (p. 35) developed a novel sensor data fusion and analysis model. By deploying multiple sensors on workpiece coatings, the model can acquire and record real-time signals of cutting force, tool vibration, and surface roughness during machining. Denkena et al. [27] (p. 35) installed accelerometers at the workpiece spindle and tailstock and arranged acoustic emission detection units on the working surface of the grinding wheel. Additionally, current sensors were configured for the spindle box drive system. The required operational signals are comprehensively collected through the above multi-sensor arrangement. Folgado et al. [28] (p. 35) proposed a novel data acquisition and monitoring system for polymer electrolyte membrane (PEM) hydrogen generators. The system adopts industrial controllers to capture operational data, which are further stored in a database via communication networks. On this basis, users can log into the system to view relevant data in real time through a web interface.

It can be concluded that traditional sensor data acquisition and transmission methods are capable of processing small-scale data in industrial control devices and equipment systems. However, the blade belt grinding robot system involves two distinct types of data, namely robot joint attitude angles and multi-source machining information generated during robot operation. Considering the differentiated characteristics of the two data categories, an OPC UA communication architecture and a cloud–edge–end database are established separately for targeted data processing and management.

This paper addresses the technical requirements for process data acquisition and visualization monitoring in blade belt grinding. It adopts a cloud–edge–device collaborative computing framework, systematically studies the construction of communication architecture, virtual modeling and client interface design, and builds a digital twin basic platform for grinding process monitoring. Taking the articulated blade grinding robot as the research prototype, this study systematically investigates communication architecture construction, virtual modeling, and client interface design and further develops a cloud–edge–end-based digital twin framework for robot-assisted blade grinding. The remainder of this paper is organized as follows. Section 2 elaborates the overall digital twin framework for the blade belt grinding robot. Section 3 establishes the corresponding digital models, including three-dimensional geometric modeling, model lightweight optimization, and kinematic modeling. Section 4 constructs a multi-source heterogeneous data acquisition and transmission system involving OPC UA-based data communication and cloud–edge–end collaborative database deployment. Section 5 develops the digital twin monitoring platform for the robot grinding process. Finally, the entire work is concluded with key findings and future research directions.

## 2. Digital Twin Overall Framework of Blade Grinding Robot

**Figure 1:**
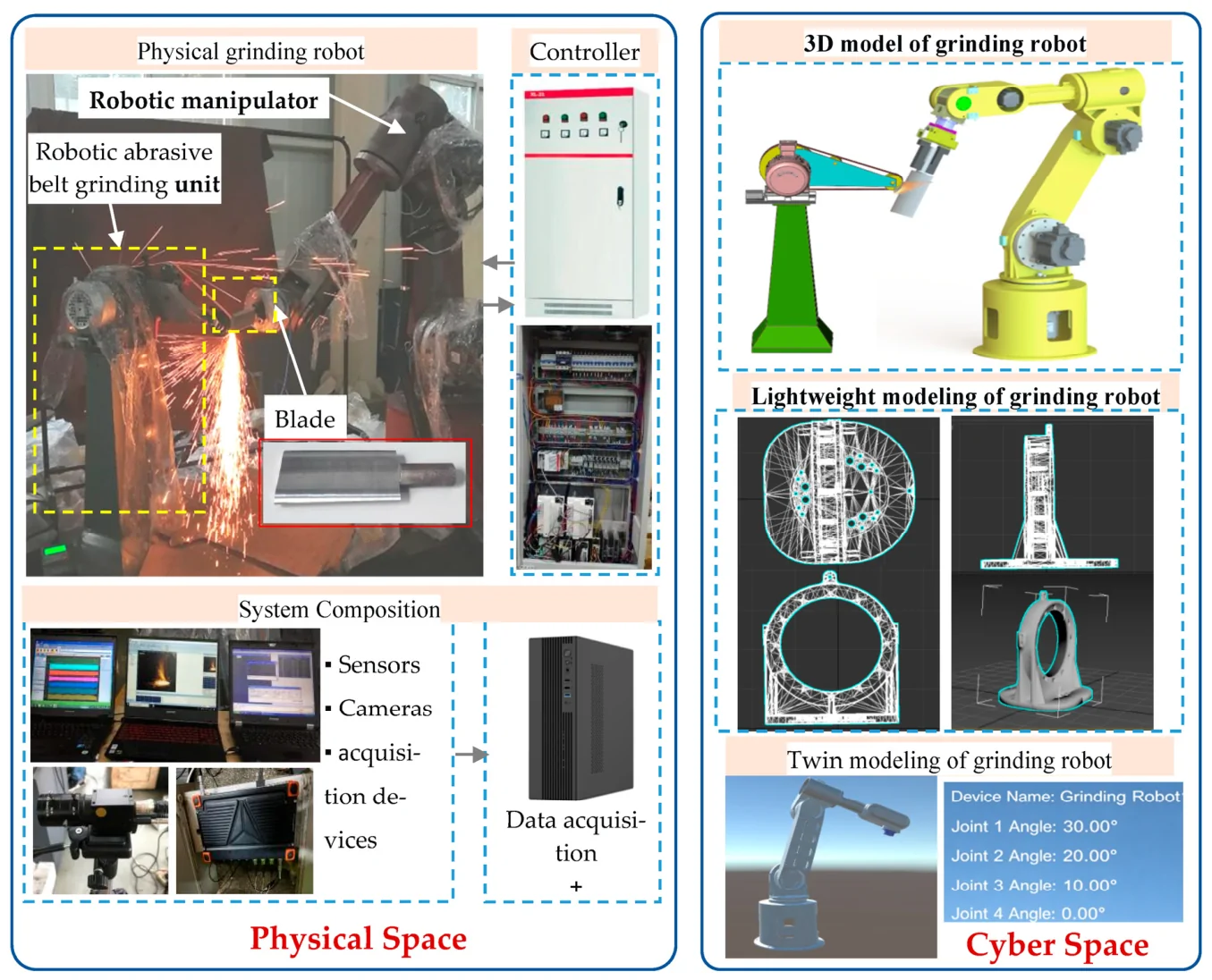
Digital twin framework for robotic belt grinding process.

This section introduces a general framework for a blade grinding robot based on a digital twin framework, as shown in Figure 1. Information collected from the physical space is used for monitoring, control, optimization, and autonomous decision making. The digital twin framework of the blade grinding robot mainly consists of two core parts: 1. physical space; 2. digital space.

The physical space is mainly composed of a physical grinding robot, a controller, and auxiliary system components. The physical grinding robot consists of a robotic manipulator, a belt grinding unit, and blade workpieces to be processed. A Beckhoff embedded PC controller (CX5140-0050, Phil, Germany) is adopted as the core control hardware, and its detailed technical parameters are listed in Table 1. The controller applies PC-based control technology based on the EtherCAT bus protocol to realize high-precision motion control. Data acquired from the physical space is transmitted to the digital space for subsequent analysis. Specifically, the controller is responsible for sending and receiving motion control commands of the robot, while extracting real-time motion-related data for the digital twin framework. In addition, the system components include multi-type sensors for collecting acoustic signals, grinding force and acceleration data, industrial CCD cameras, and professional data acquisition devices. The collected multi-source data is acquired and preprocessed by the server to support subsequent digital twin analysis.

The construction of the digital space follows a standardized modeling procedure. Firstly, a three-dimensional geometric model of the grinding robot is established and further optimized through lightweight processing to obtain a simplified high-efficiency model. On this basis, kinematic and dynamic modeling is performed to construct the complete digital twin framework model of the grinding robot. In the digital space, the entire belt grinding process can be quantitatively analyzed based on real-time data collected from the robot controller and multi-source data acquisition units. The database stores all physical sensing and operational data, which supports cloud computing, visual rendering, dynamic simulation, and intelligent data analysis, thereby providing a reliable data basis for intelligent decision making of the grinding system.

For the communication network, the OPC UA protocol architecture is deployed to realize bidirectional data transmission and instruction interaction between the edge terminal and the central cloud. Physical equipment, including the belt grinding robot and CX5140 controller, is connected to the OPC UA server through the industrial connection platform KEPServer EX. The OPC UA client acquires real-time data from the server to support upper-level autonomous decision making. Furthermore, intelligent algorithms such as machine learning and convolutional neural networks are adopted for model comparison and parameter optimization, realizing adaptive process adjustment and visual monitoring of the blade belt grinding process.

## 3. Digital Model of Blade Belt Grinding Robot

### 3.1. 3D Virtual Modeling of Blade Belt Grinding Robot

**Figure 2:**
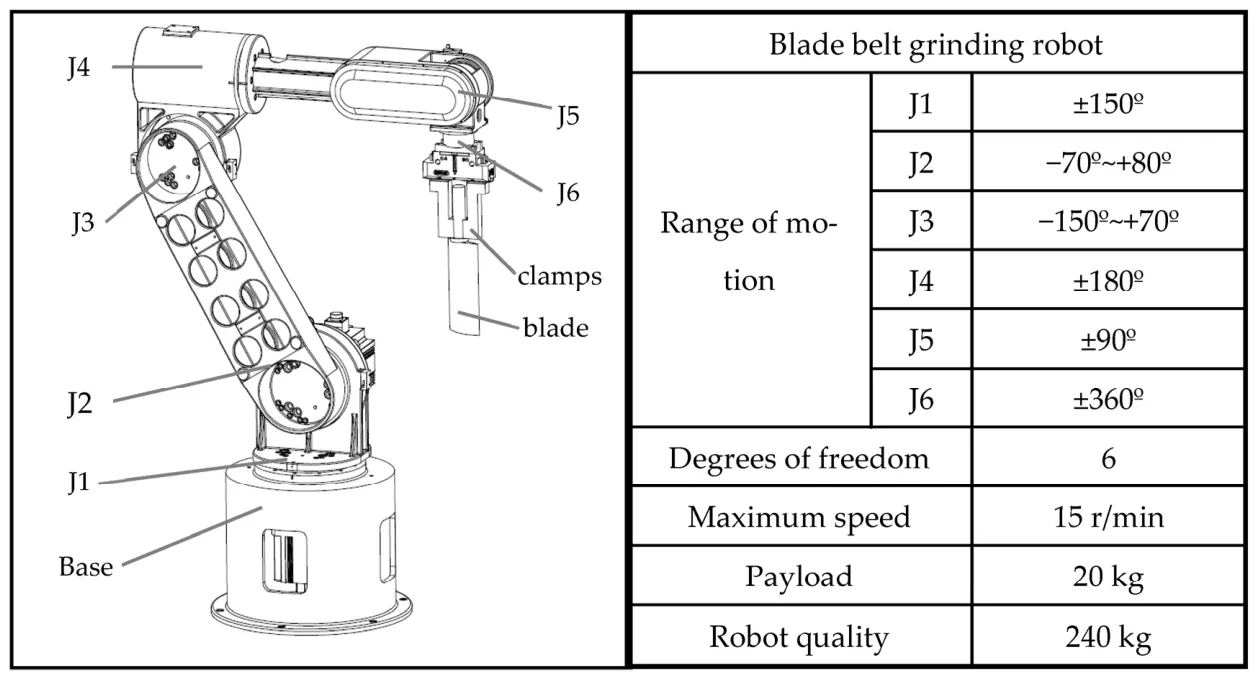
Basic parameters of blade belt grinding robot.

The blade belt grinding robot adopted in this study is equipped with six movable joints, including the base rotation joint (J1), lower boom pitch joint (J2), upper boom pitch joint (J3), arm swing joint (J4), wrist pitch joint (J5), and wrist rotation joint (J6). An independent gripper mechanism is installed at the robot end-effector. Through the cooperative motion of multiple joints, the end tool can reach arbitrary positions and postures within the working space, enabling high-flexibility grinding processing for complex blade surfaces. The basic structural parameters of the robot are presented in Figure 2.

**Figure 3:**
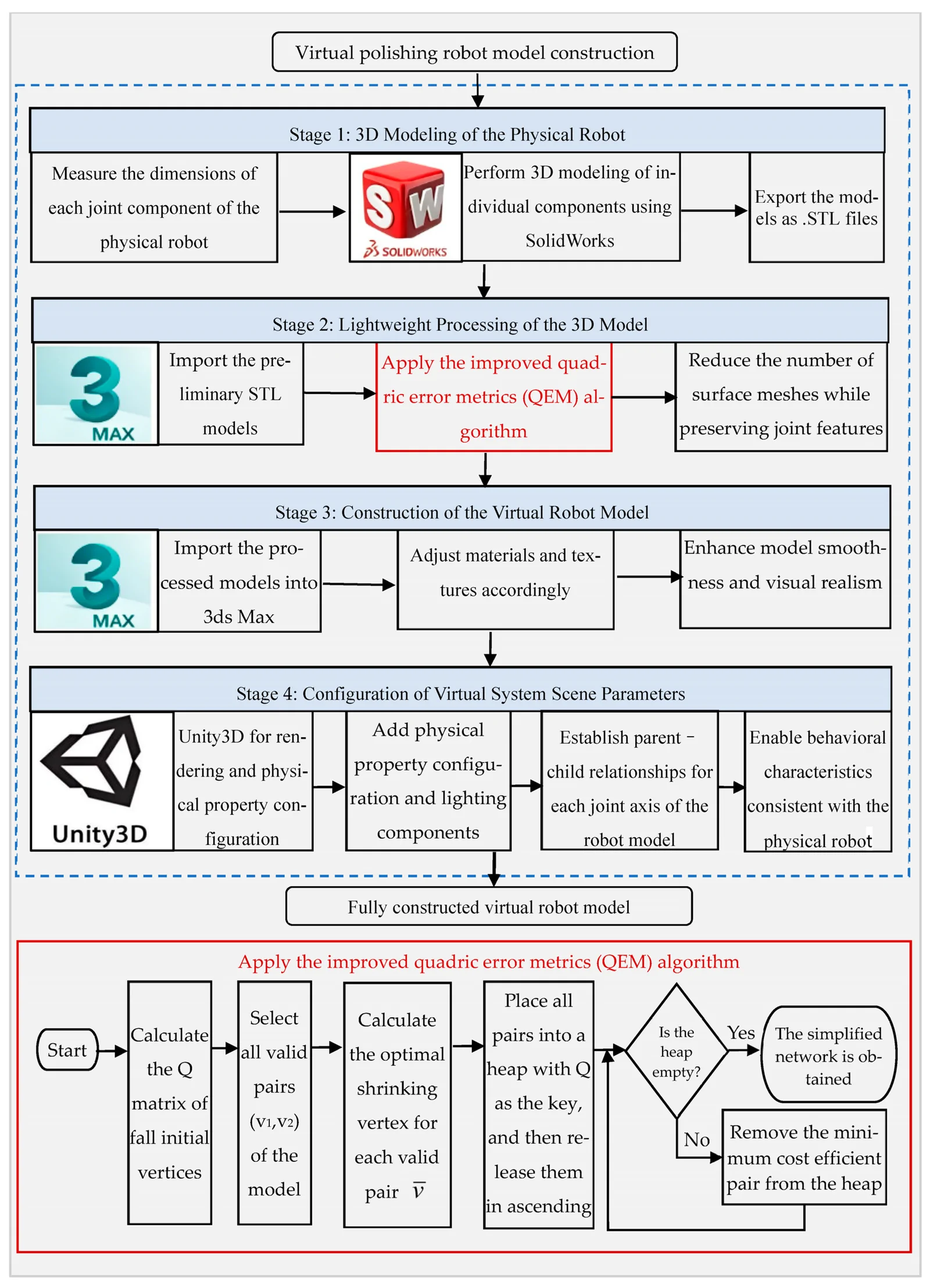
Modeling process of virtual robots.

The three-dimensional model serves as the geometric foundation for the construction of the digital twin framework. In the development of the virtual robot model, accurate mapping of physical entities and iterative optimization of the model are essential to guarantee the consistency and reliability of the digital twin system. The construction of the virtual robot model mainly includes three stages: the establishment of a three-dimensional model of the physical robot, lightweight optimization of the three-dimensional model, and the final construction of the robot virtual twin model. The specific implementation process is illustrated in Figure 3.

**3D Modeling of the Physical Robot**: The dimensions of each joint component of the physical robot are measured, and three-dimensional modeling of the individual parts is completed using SolidWorks software. After the part models are created, they are converted into the corresponding .STL file format and exported, yielding preliminary 3D models of the robot components.

**Lightweight Processing** of 3D Models: The preliminary 3D models of the robot components are subjected to lightweight processing using an improved quadric error metrics (QEM) algorithm. This approach reduces the number of mesh faces while preserving the joint features of the models, thereby alleviating the computational burden on the computer.

**Construction of the Virtual Robot Model**: The processed 3D models of the robot components are imported into 3ds Max software, where appropriate materials and textures are applied. This enhances both the visual realism and rendering smoothness of the virtual robot model.

**Configuration of Virtual System Scene Parameters**: Finally, the robot model is imported into Unity 3D 2023 software for rendering and physical property configuration. Appropriate lighting components are added to enhance the rendering quality. For each joint axis of the robot model, a corresponding parent–child relationship is established, enabling the virtual model to exhibit kinematic behavior identical to that of the physical robot.

### 3.2. Lightweight Model Processing

3D models are typically represented by triangular meshes; the denser the mesh, the finer the geometric details. When the model is far from the viewpoint or not a visual focal point, rendering with high-density meshes incurs unnecessary computational overhead. Therefore, mesh simplification techniques can be used in such cases, eliminating the need to retain fine details. Mesh simplification mainly falls into two categories: redrawing and deletion. Redrawing destroys the topology, easily alters the appearance, and has poor results; and it has wider applications. Deletion methods can be further divided into vertex deletion, edge collapsing, and triangular face collapsing. Among these, the edge collapsing algorithm strikes a good balance between efficiency and accuracy, so this study chooses this method to simplify the 3D model of the grinding robot.

Garland’s quadric error metrics (QEM) algorithm is currently the most commonly used edge folding algorithm [29] (p. 35). This algorithm uses the square of the distance from the new vertex to the associated triangle as the error criterion, effectively measuring simplification errors. The QEM algorithm flowchart is shown in the red area of Figure 3. The principle is as follows:

For two vertices $v_{1}$ and $v_{2}$, collapse them into a new vertex $\bar{v}$. Let $plane(v_{i})$ denote the original set of triangles associated with vertex $v_{i}$, then the optimization objective is to minimize the following error function:

(1) $$\Delta (v)=\sum_{p\in plane(v)}{\left(p^{T}v\right)}^{2}$$

Let the expression for plane p be ax+by+cz+d=0, where $a^{2}+b^{2}+c^{2}=1$ and d is a constant. Let $v={\left[x,y,z,1\right]}^{T}$ and $p={\left[a,b,c,d\right]}^{T}$, then we can obtain:

(2) $${\left(p^{T}v\right)}^{2}=0$$

In the formula, the 4 × 4 symmetric matrix $K_{p}=pp^{T}$ is defined as follows:

(3) $$K_{p}=pp^{T}=\left[\begin{array}{cccc} a^{2} & ab & ac & ad\\ ab & b^{2} & bc & bd\\ ac & bc & c^{2} & cd\\ ad & bd & cd & d^{2} \end{array}\right]$$

By substituting Equation (2) into Equation (1), we can obtain:

(4) $$\Delta (v)=\sum_{p\in plane(v)}(v^{T}K_{p}v)=v^{T}(\sum_{p\in plane(v)}K_{p})v$$

Let $Q=\sum_{p\in plane(v_{i})}K_{p}$, then

(5) $$\Delta (v)=v^{T}Qv$$

Let $Q=Q_{1}+Q_{2}$ be the error matrix associated with vertices $v_{1}$ and $v_{2}$; the cost of edge collapse is then expressed as:

(6) $$\Delta (\bar{v})={\bar{v}}^{T}(Q_{1}+Q_{2})\bar{v}$$

To determine the new vertex $\bar{v}$ that minimizes the error, the partial derivative of Equation (6) is taken and set to zero:

(7) $$\frac{\partial \Delta \bar{v}}{\partial \bar{v}}=2(Q_{1}+Q_{2})\bar{v}=0$$

Let $Q=Q_{1}+Q_{2}$, then:

(8) $$Q\bar{v}=0$$

To express Q and $\bar{v}$ in block form, let:

(9) $$Q=\left[\begin{array}{cc} A & b\\ b^{T} & c \end{array}\right],\;\bar{v}=\left[\begin{array}{c} x\\ 1 \end{array}\right]$$

The determination of the optimal new vertex is then categorized into the following two scenarios:

(1) When *A* is non-singular, the optimal new vertex is $\bar{v}=-A^{-1}b$, which yields the minimum error.
(2) When *A* is singular, the two edge endpoints and the midpoint of the edge are taken as candidate vertices. The cost Δ(v) is evaluated for each candidate, and the vertex corresponding to the smallest error is selected as the optimal one.

**Figure 4:**
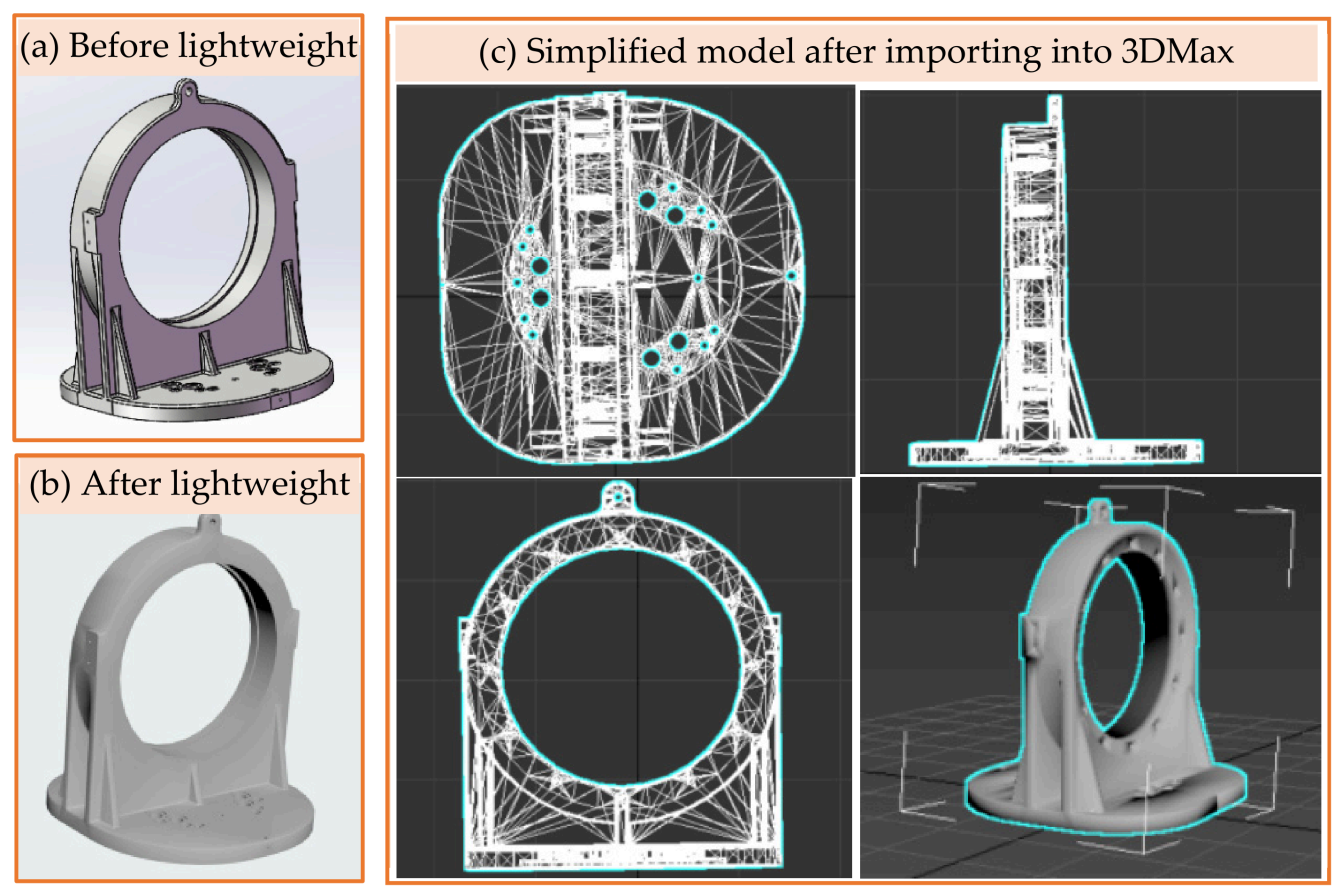
The simplified joint axis model.

Applying the QEM algorithm described above, the key components of the grinding robot are simplified. Figure 4 illustrates a representative joint of the robot before and after model lightweighting.

As can be seen from the comparison of Figure 4a,b above, although the surface finish of the joint axis model is reduced after the lightweighting process, it still retains key details. After importing the simplified model into 3D Max 2025 software, as shown in Figure 4c, the model as a whole does not show any distortion.

Using a similar method, the other joints of the robot were simplified. The numbers of triangular facets before and after simplification are shown in Table 2 below.

As shown in the Table 2, the edge collapse algorithm based on quadric error metrics (QEM) achieves significant mesh simplification across all joint axes. Among them, Joint Axis 2 possesses the largest number of original triangular facets (25,080), which is reduced to 2483 after simplification—a reduction of approximately 90.1%, representing the greatest absolute reduction among the five joint axes. Joint Axis 5 has the fewest original facets (7796), which are reduced to 757, corresponding to a reduction ratio of about 90.3%. The simplification ratios of all joint axes range from 89.4% to 90.6%, demonstrating a high level of consistency and indicating that the QEM algorithm exhibits stable simplification performance across models of varying geometric complexity. With a facet reduction rate of approximately 90% while maintaining visual acceptability, the proposed simplification strategy provides a reliable model foundation for subsequent real-time rendering and interaction in the system.

### 3.3. Kinematic Modeling of the Grinding Robot

To achieve precise positioning and attitude control of the end-effector of the blade grinding robot in three-dimensional space, it is necessary to establish its kinematic model. In this paper, the modified Denavit–Hartenberg (D-H) parameter method is adopted. Following the standard convention for coordinate frame assignment, the geometric and kinematic relationships between adjacent links are systematically described. Compared with the conventional D-H method, the modified approach incorporates adaptive adjustments in coordinate frame allocation and parameter selection, which effectively reduces modeling errors caused by redundant parameters of adjacent joints, making it particularly suitable for high-precision kinematic modeling applications [30,31] (p. 35).

**Figure 5:**
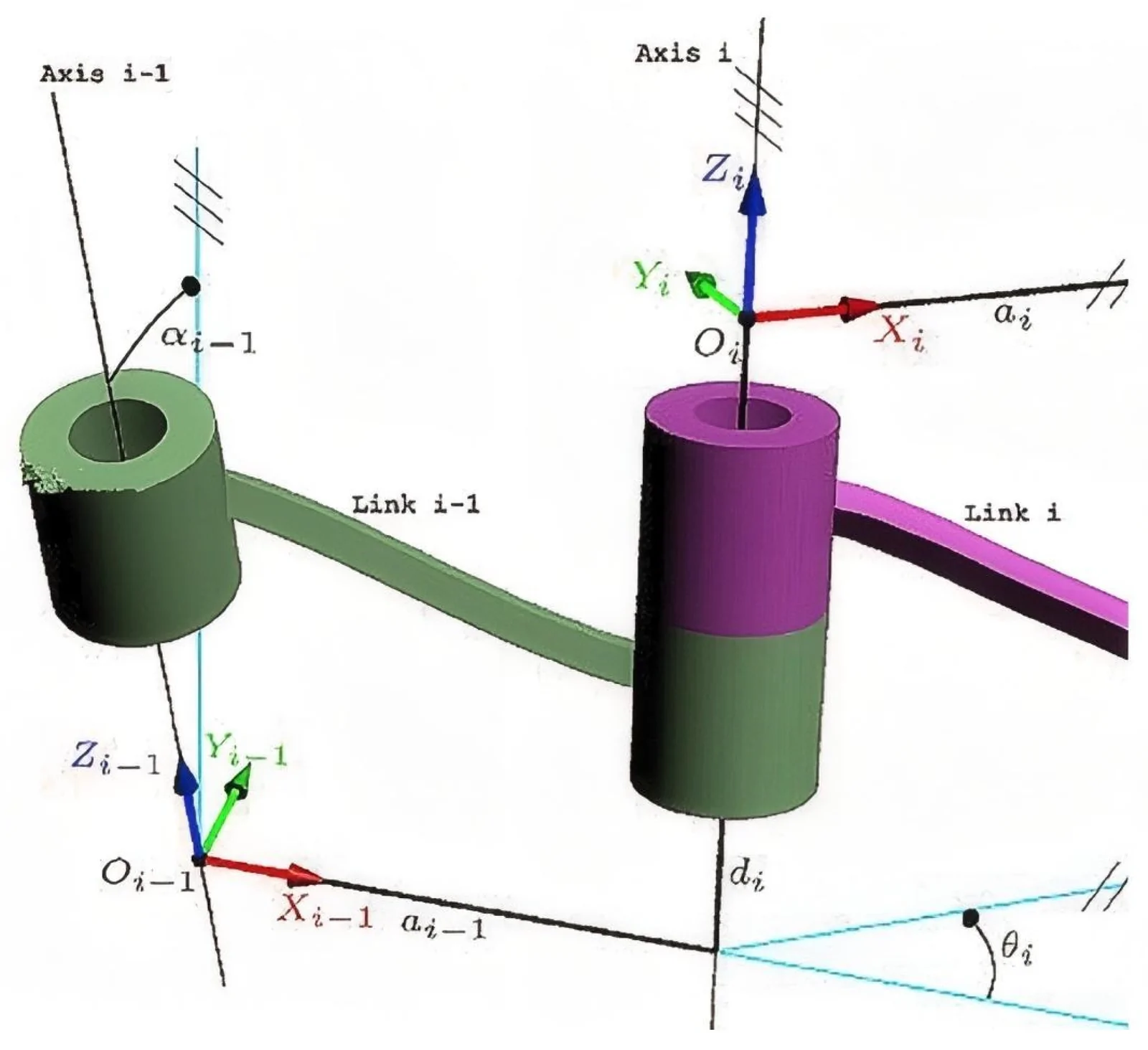
Modified D-H method modeling diagram.

Based on the modified Denavit–Hartenberg (D-H) method, the coordinate frames of each robot joint are established, as illustrated in Figure 5. Four parameters—namely $a_{i-1},\alpha_{i-1},d_{i},\theta_{i}$—are required for the modeling process, and their specific definitions are summarized in Table 3.

The transformation matrix from coordinate system $O_{i-1}$ to coordinate system $O_{i}$ can be obtained as follows:

(10) Tii−1=Rotxi−1(αi−1)×Transxi−1(ai−1)×Rotzi(θi)×Transzi(di)

The expanded form is given as follows:

(11) Tii−1=cosθi−sinθi0ai−1sinθicosαi−1cosθicosαi−1−sinαi−1−disinαi−1sinθisinαi−1cosθisinαi−1cosαi−1dicosαi−10001

#### 3.3.1. Forward Kinematics Analysis

**Figure 6:**
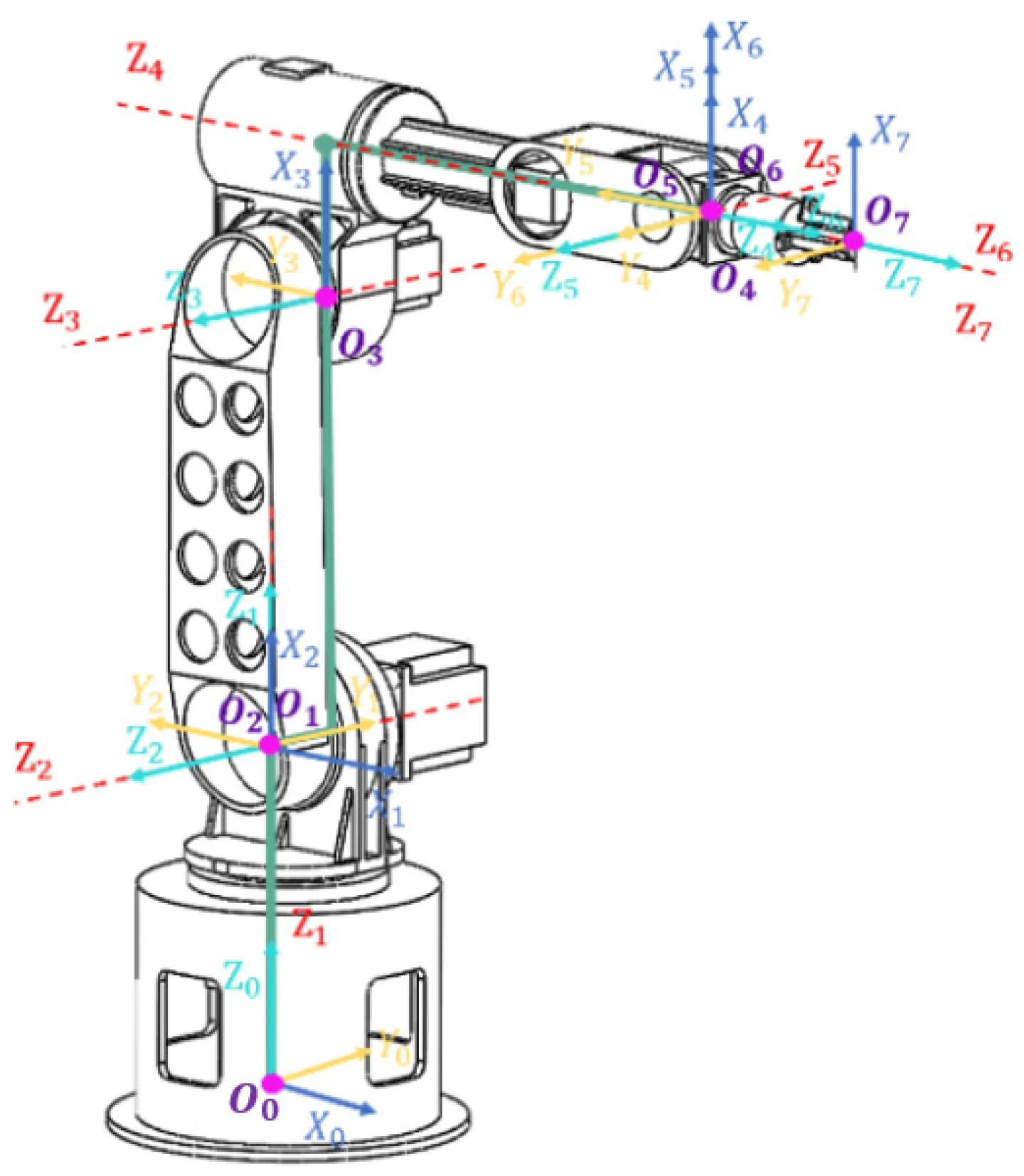
Robot joint reference coordinate system.

The grinding robot is a six-degree-of-freedom articulated robot. According to the establishment rules of the modified Denavit–Hartenberg (D-H) method, corresponding coordinate frames need to be assigned to all joint links. First, the base coordinate frame is established at the center of the base and denoted as $O_{0}$. Then, the rotation centers of the six joint axes are sequentially denoted as $O_{1},O_{2},O_{3},O_{4},O_{5},O_{6}$ and $O_{7}$. Since joints $O_{1}$ and $O_{2}$ are serially connected revolute joints, their coordinate frames are set to coincide. Furthermore, since the axes of joints $O_{4},O_{5}$, and $O_{6}$ intersect at a common point, their coordinate frames are all placed at this point (i.e., the telecentric point) for computational convenience. The resulting simplified model after the above coordinate frame assignment is shown in Figure 6.

Based on the reference coordinate frame model established above, the relevant parameters of each robot joint can be determined. Using the modified Denavit–Hartenberg (D-H) method, the parameters for each joint are listed in Table 4 below.

The final transformation matrices are given as Equation (12).

(12) $$A_{1}=\left[\begin{array}{cccc} c\theta_{1} & -s\theta_{1} & 0 & 0\\ s\theta_{1} & c\theta_{1} & 0 & 0\\ 0 & 0 & 1 & d_{1}\\ 0 & 0 & 0 & 1 \end{array}\right]\;A_{2}=\left[\begin{array}{cccc} c\theta_{2} & -s\theta_{2} & 0 & 0\\ 0 & 0 & -1 & 0\\ s\theta_{2} & c\theta_{2} & 0 & 0\\ 0 & 0 & 0 & 1 \end{array}\right]\;A_{3}=\left[\begin{array}{cccc} c\theta_{3} & -s\theta_{3} & 0 & a_{2}\\ s\theta_{3} & c\theta_{3} & 0 & 0\\ 0 & 0 & 1 & -d_{3}\\ 0 & 0 & 0 & 1 \end{array}\right]\;A_{4}=\left[\begin{array}{cccc} c\theta_{4} & -s\theta_{4} & 0 & a_{3}\\ 0 & 0 & -1 & -d_{4}\\ s\theta_{4} & c\theta_{4} & 0 & 0\\ 0 & 0 & 0 & 1 \end{array}\right]\;A_{5}=\left[\begin{array}{cccc} c\theta_{5} & -s\theta_{5} & 0 & 0\\ 0 & 0 & 1 & 0\\ -s\theta_{5} & -c\theta_{5} & 0 & 0\\ 0 & 0 & 0 & 1 \end{array}\right]\;A_{6}=\left[\begin{array}{cccc} c\theta_{6} & -s\theta_{6} & 0 & 0\\ 0 & 0 & -1 & 0\\ s\theta_{6} & c\theta_{6} & 0 & 0\\ 0 & 0 & 0 & 1 \end{array}\right]\;A_{7}=\left[\begin{array}{cccc} 1 & 0 & 0 & 0\\ 0 & 1 & 0 & 0\\ 0 & 0 & 1 & d_{7}\\ 0 & 0 & 0 & 1 \end{array}\right]$$

Among the joint variables: $s\theta_{i}$ is $\sin\left(\theta_{i}\right)$, $c\theta_{i}$ is $\cos\left(\theta_{i}\right)$.

Based on the above transformation matrices of each joint, the pose matrix of the robot end-effector can be obtained as follows:

To verify the correctness of the forward kinematics modeling results, substituting the joint variable data of the robot’s initial posture $\theta_{1}=0{}^{\circ}$, $\theta_{2}=90{}^{\circ}$, $\theta_{3}=0{}^{\circ}$, $\theta_{4}=0{}^{\circ}$, $\theta_{5}=0{}^{\circ}$, $\theta_{6}=0{}^{\circ}$, as shown in Equation (13).

(13) T70=001d4+d70−10d3100a2+a3+d10001=0019900−10−4610015480001

After comparing with the actual end-effector posture of the robot under the initial posture, it is found that the results are in good agreement with the calculated ones, indicating that the forward kinematics analysis of the robot is reasonable.

#### 3.3.2. Inverse Kinematics Analysis

The inverse kinematics of the robot is the reverse solution of the forward kinematics equations. Given the known position and orientation of the robot end-effector, the joint angles required for the robot to reach the specified pose are inversely calculated. The commonly used mathematical methods for solving inverse kinematics equations include the inverse transformation method, the geometric method, etc. The inverse transformation method is adopted to carry out the inverse kinematics analysis of the grinding robot. The robot kinematic equations are shown in Equation (14).

(14) T60=nxoxaxpxnyoyaypynzozazpz0001=T10θ1T21θ2T32θ3T43θ4T54θ5T65θ6

To solve for the Joint Axis 1 angle $\theta_{1}$, multiply both sides of Equation (14) by T−110^1^, yielding:

The solution procedure for the remaining five joint angles is analogous to that for $\theta_{1}$, and thus the detailed derivation is omitted here for brevity. The derived joint angle solutions are summarized in Table 5.

In theory, a six-degree-of-freedom robot possesses eight sets of inverse kinematic solutions. However, in practical operation, due to workspace constraints, not all of these solutions are physically realizable. Therefore, in robot motion control, optimal solutions are typically selected based on criteria such as shortest path or minimum time. Since this study requires precise monitoring of each robot joint, the optimal solution is determined according to the principle of position optimality.

In Table 5: $p_{x}=\rho_{1}\cos\phi ;\;p_{y}=\rho_{1}\sin\phi$; $\rho_{2}=\sqrt{a_{3}^{2}+d_{4}^{2}}$;

(15) $$k=\frac{{\left(p_{z}-d_{1}\right)}^{2}+p_{x}^{2}+p_{y}^{2}-a_{2}^{2}-a_{3}^{2}-d_{3}^{2}-d_{4}^{2}}{2a_{2}}$$

## 4. Multi-Source Heterogeneous Data Acquisition and Transmission Architecture

To enable real-time monitoring and data analysis of the grinding robot’s operational status and machining process, two types of heterogeneous data are addressed: joint posture angles (as strong real-time data) and machining process information (including spark images, grinding sound, grinding force, and acceleration). Accordingly, an OPC UA-based data acquisition and transmission architecture and a cloud–edge–end collaborative database system are respectively constructed.

### 4.1. OPC UA-Based Heterogeneous Data Acquisition and Transmission Architecture

#### 4.1.1. Data Generation and Acquisition During the Machining Process

Machining process data are critical to the fidelity of the digital twin framework model. During the grinding process, key process parameters—including abrasive grain size, belt width, abrasive material, workpiece material, belt speed, feed rate, theoretical grinding depth, and applied pressure—are known a priori and can be stored in the database. However, due to the inherent complexity of the grinding process and the inevitable wear of the abrasive belt, the material removal capability of the abrasive grains gradually deteriorates over time. This leads to a deviation in the equivalent relationship between grinding force and material removal volume, making it difficult to accurately obtain the actual material removal rate (MRR) through theoretical models alone.

Visual and auditory information serve as the most direct indicators for sensing the material removal rate. Experienced operators typically assess the grinding removal rate by observing the sparks generated during the process and listening to the grinding sounds. Accordingly, in the data transmission framework established in this study, spark images and acoustic signals are collected, and the real-time variation of the actual material removal rate is treated as a dynamic variable fed into the database. This design aims to enhance the accuracy and reliability of the digital twin model.

**Figure 7:**
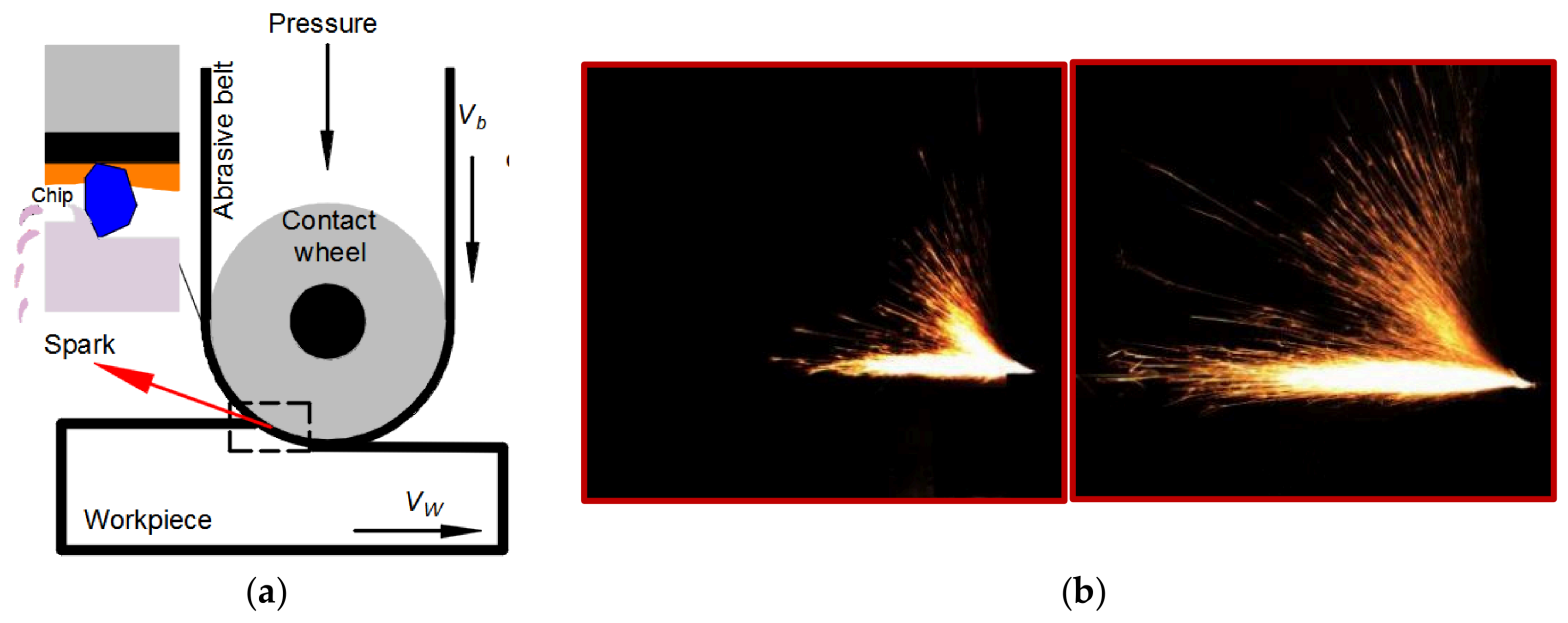
Generation of grinding sparks and the collected spark images. (**a**) The generation mechanism of spark images. (**b**) Acquired spark images.

Grinding sparks are formed when chips generated during the grinding process carry substantial heat and undergo oxidation and combustion in the air, as illustrated in Figure 7a. The quantity of grinding sparks is directly proportional to the volume of chips produced; therefore, grinding sparks can serve as a direct indicator of the material removal rate (MRR). Grinding equipment is typically enclosed, and the relatively dim ambient light within the working area creates a pronounced contrast with the bright spark region, making it convenient to capture spark images. Since the grinding spark phenomenon is inherently dynamic, the acquisition system must support automatic image capture and storage functions throughout the grinding process. In this study, an HT-GE200GC/GE200GM industrial CMOS camera is employed to capture spark images during grinding. Representative spark images corresponding to different material removal rates are shown in Figure 7b. The material removal rates from left to right are 0.287 g/(mm·min) and 0.190 g/(mm·min), respectively. All acquired image data are stored in the database in a predefined fixed format.

**Figure 8:**
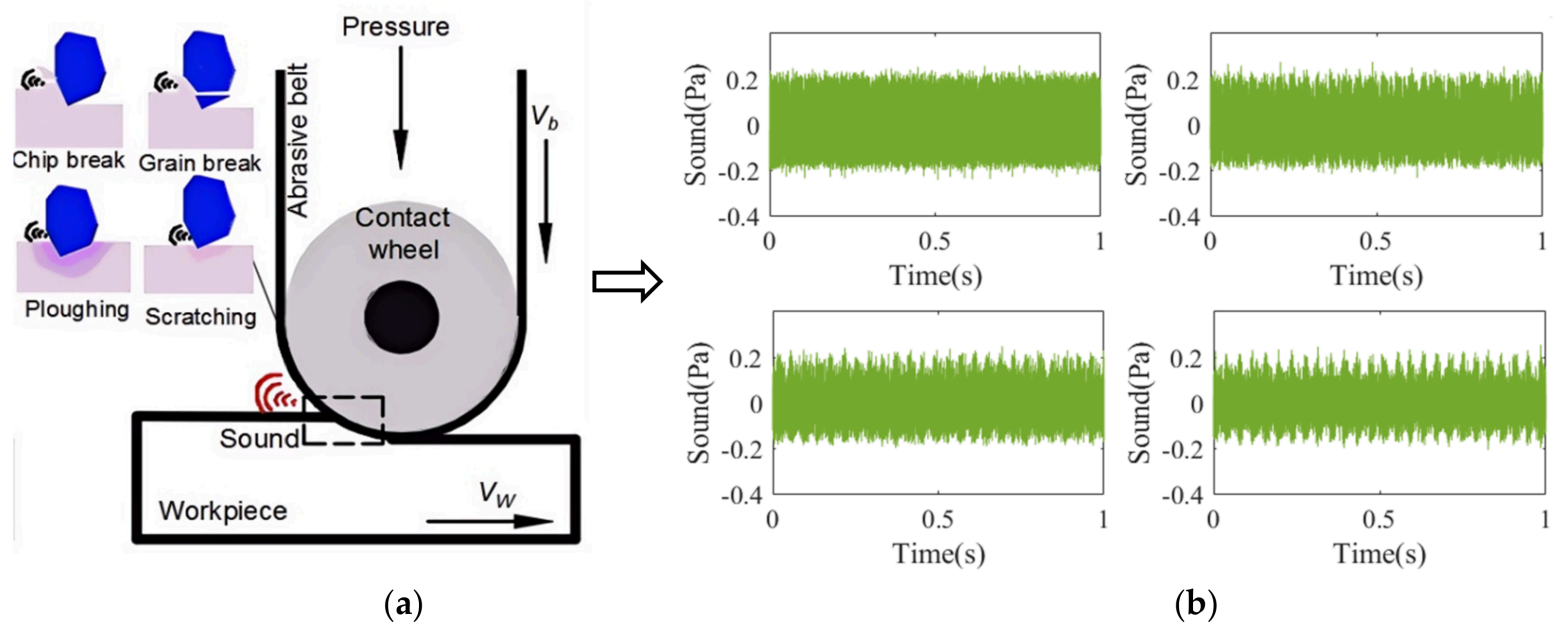
Generation of grinding sound and collected sound signals. (**a**) Mechanism of grinding sound generation. (**b**) Collected grinding sound signals.

During material removal, the grinding process generates acoustic emissions resulting from various mechanical interactions, including sliding friction, ploughing forces, cutting forces, and air pressure fluctuations induced by workpiece vibration due to plastic deformation. A schematic illustration of the sound formation mechanism associated with the material removal process is presented in Figure 8a. Under different operating conditions—such as varying grinding parameters and workpiece properties—the magnitudes of sliding friction, ploughing forces, cutting forces, and plastic deformation differ, thereby producing distinct grinding sounds.

In addition to the effective acoustic signals related to material removal, the acquired grinding sound also contains mechanical noise from the machine tool, the belt drive motor, and structural vibrations, as well as background noise from the surrounding environment. The background noise can be effectively suppressed through appropriate filtering techniques. In this study, an MP40 integrated microphone is employed as the acoustic sensor. A DEWESoft data acquisition system is used to convert the captured analog signals into digital format for storage. The acquired acoustic signals corresponding to different material removal rates are shown in Figure 8b. From left to right and top to bottom, the four waveforms in Figure 8b correspond to material removal rates of 0.287 g/(mm·min), 0.128 g/(mm·min), 0.190 g/(mm·min), and 0.0819 g/(mm·min), respectively. All acoustic data are stored in the database in a predefined fixed format.

#### 4.1.2. Heterogeneous Data Transmission Architecture Based on OPC UA

**Figure 9:**
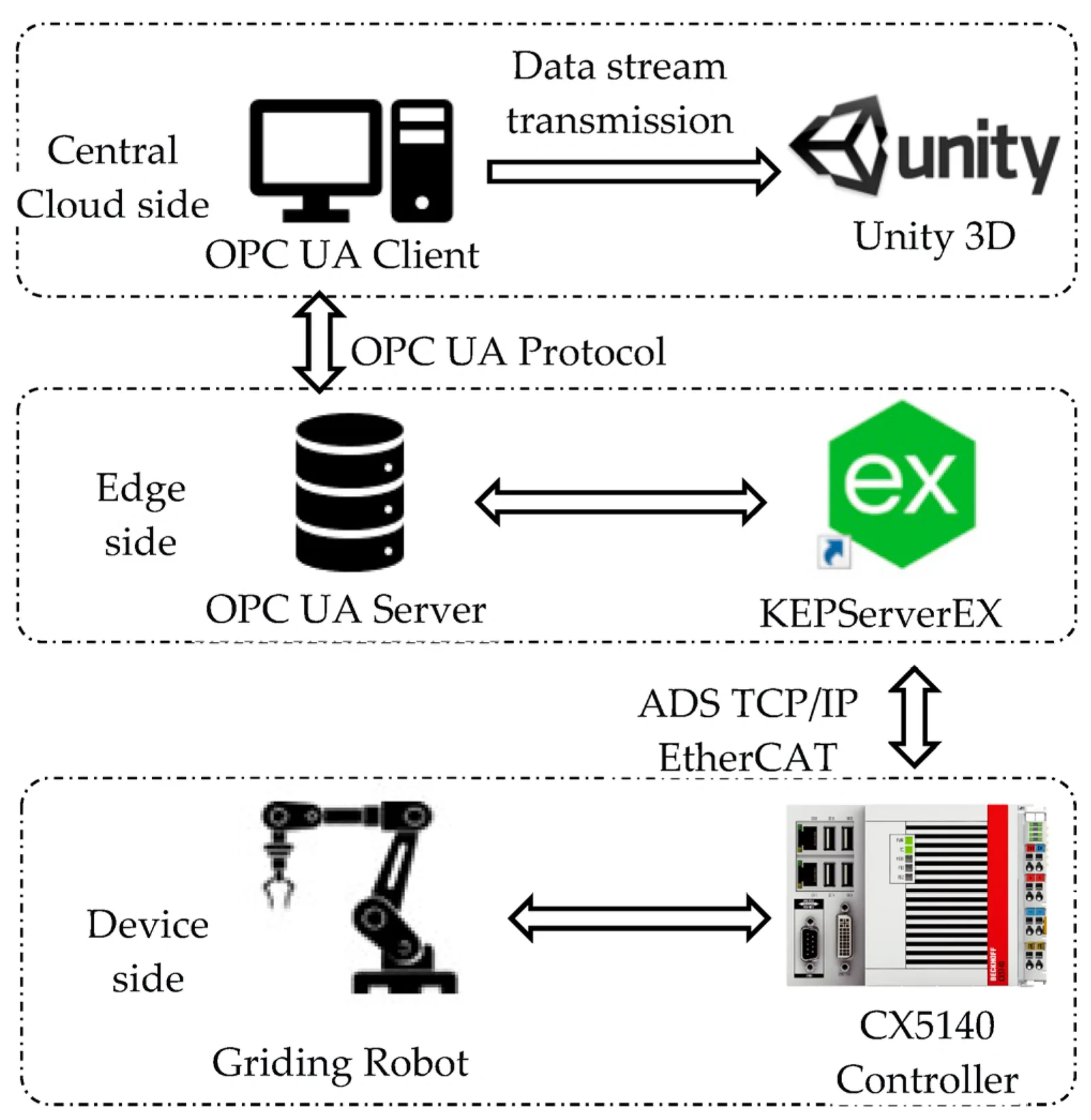
Data acquisition and transmission architecture based on OPC UA.

In this study, a three-layer data acquisition and transmission architecture is proposed, as illustrated in Figure 9. The architecture consists of three tiers: the device layer, the edge layer, and the central cloud layer. The device layer comprises a six-degree-of-freedom grinding robot and a Beckhoff CX5140 controller (Phil, Germany). At the edge layer, an OPC UA server is deployed, which is responsible for acquiring joint posture data from the device layer, performing protocol conversion from Automation Device Specification (ADS) to OPC UA, and providing a unified upstream communication interface. At the central cloud layer, an OPC UA client is deployed to establish a connection with the edge server and stream the posture data to the Unity 3D monitoring engine in real time [32] (p. 35).

### 4.2. Implementation of OPC UA Data Access on the Device Layer

To enable data interaction between the OPC UA server and the device layer, it is essential to ensure that the device-side configuration provides a data communication interface with OPC UA protocol support. The grinding robot on the device side in this study is built upon the Beckhoff control architecture. Therefore, the configuration of the OPC UA communication channel is demonstrated below using Beckhoff TwinCAT 3 PLC (Phil, Germany) as an example.

In the TwinCAT 3 PLC development environment, the visibility and access permissions (read-only or read/write) of PLC variables within the OPC UA address space are defined by configuring attributes such as OPC.UA.DA for each variable. The specific configuration methods for these attributes are summarized in Table 6.

To verify the validity of the communication channel, a readable/writable variable “nCounter” and a read-only variable “nNumber” were defined in the TwinCAT PLC, and read verification was performed using the TwinCAT OPC UA (Phil, Germany) Sample Client.

### 4.3. OPC UA Server Deployment at the Edge with Multi-Protocol Data Exchange

In this study, KEPServerEX was adopted as the OPC UA server solution. The server construction process consisted of three key steps: channel configuration (selecting the Beckhoff TwinCAT protocol), device configuration (specifying the TwinCAT PLC model), and node configuration (defining node name, address, data type, and a scan rate of 100 ms). Upon completion of the configuration, the data acquisition functionality was verified using the built-in client of KEPServerEX.

To address the data silo problem caused by the coexistence of multi-brand industrial devices, this study leverages the Advanced Tags functionality of KEPServerEX, particularly the Link Tag, to enable data exchange between devices using different protocols. Taking Beckhoff TwinCAT PLC (ADS protocol) and a Modbus virtual device (Modbus protocol) as an example, a Link Tag is created on the server side to achieve data mapping and synchronization between the two device nodes, thereby eliminating redundant data transmission at the client side. By monitoring the value changes of the “nCounter” node in the TwinCAT PLC, it is demonstrated that the value written to the Modbus device can be synchronously reflected in the TwinCAT PLC node in real time, confirming the feasibility of the cross-protocol data interaction mechanism.

### 4.4. Design of Key Functional Modules for the OPC UA Client

**Figure 10:**
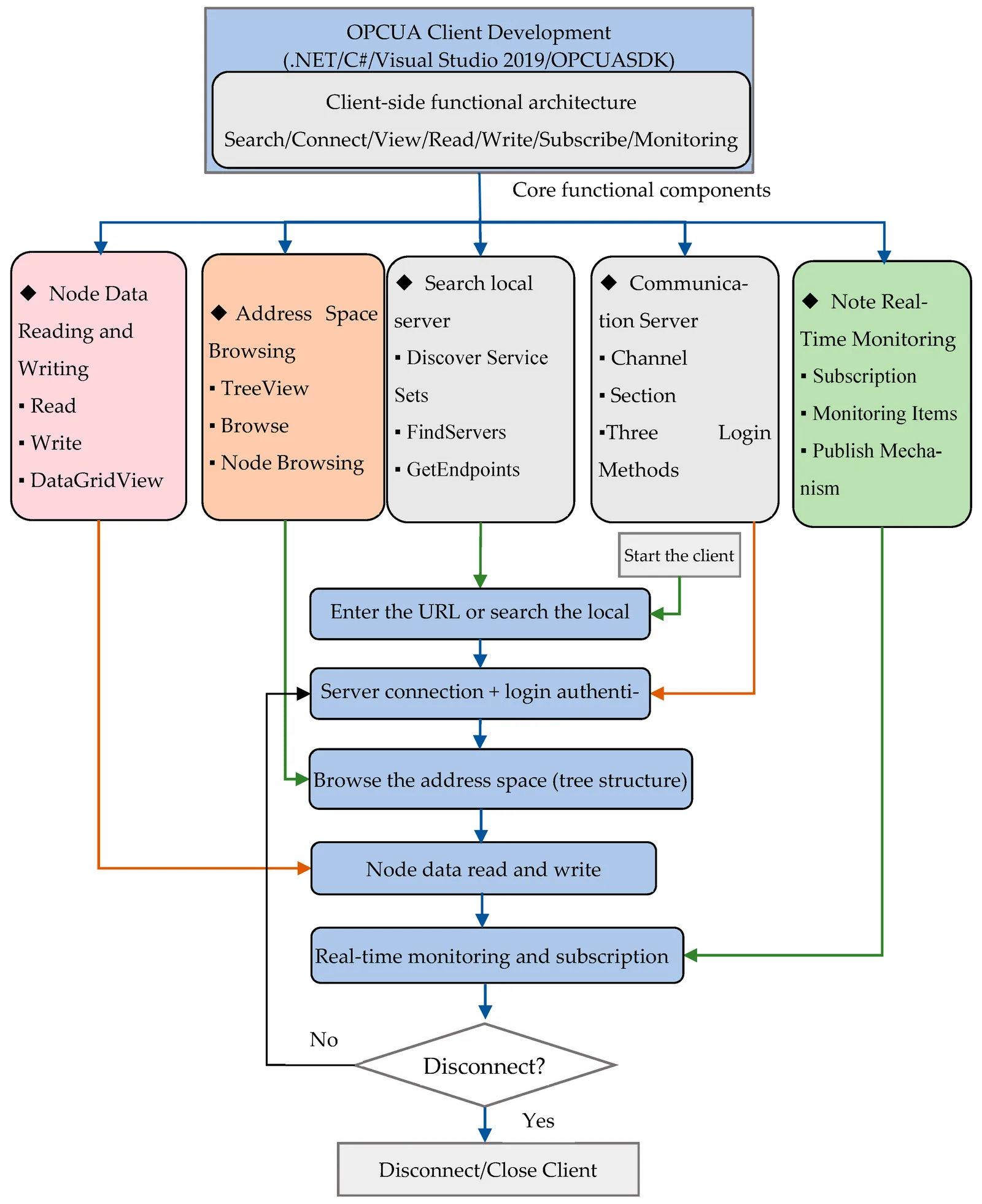
Basic architecture and operation flowchart of the OPC UA client.

To meet the requirements of real-time data acquisition and interaction for the grinding robot monitoring platform, a complete OPC UA client system was designed and implemented based on the .NET/C#/Visual Studio 2019 platform and the OPC UA Foundation SDK. The system strictly complies with the OPC UA standard specifications and incorporates core functionalities including server discovery, secure connection establishment, address space browsing, node data read/write, and real-time subscription and monitoring. The overall functional architecture and operational workflow are illustrated in Figure 10.

As shown in Figure 10, the standard operational workflow of the OPC UA client can be summarized as follows: start the client→enter the server URL or search for local servers→establish a connection and perform login authentication→browse the address space (in a tree structure)→execute node data read/write operations→enable real-time subscription and monitoring→disconnect or close the client.

In terms of functional architecture, the client is logically divided into five core components: local server discovery component, communication server component, address space browsing component, node data read/write component, and node data real-time monitoring component. The functions of each component are described as follows:

The local server discovery component is implemented based on the OPC UA Discovery Service Sets. By invoking the “FindServers” and “GetEndpoints” services, the client can automatically discover registered OPC UA servers within the current network environment, which is particularly suitable for complex scenarios such as multi-robot collaborative operations.

**Communication Server Component:** This component supports connection establishment either by entering the server URL or by selecting a discovered server. During session establishment, it provides three authentication methods (anonymous login, username/password login, and certificate login) in accordance with the server-configured security policies (including None, Sign, and Sign & Encrypt), thereby ensuring the security of the communication channel.

**Address Space Browsing Component:** Upon successful connection, this component invokes the “Browse” service to parse the node hierarchy of the server address space and displays it in a tree view (TreeView), allowing users to navigate and access nodes intuitively.

**Node Data Read/Write Component:** Based on the OPC UA standard “Read” and “Write” services, this component implements reading and writing of node data. Data information (including name, value, data type, access rights, etc.) is presented in a DataGridView control, with support for online modification of writable nodes.

**Figure 11:**
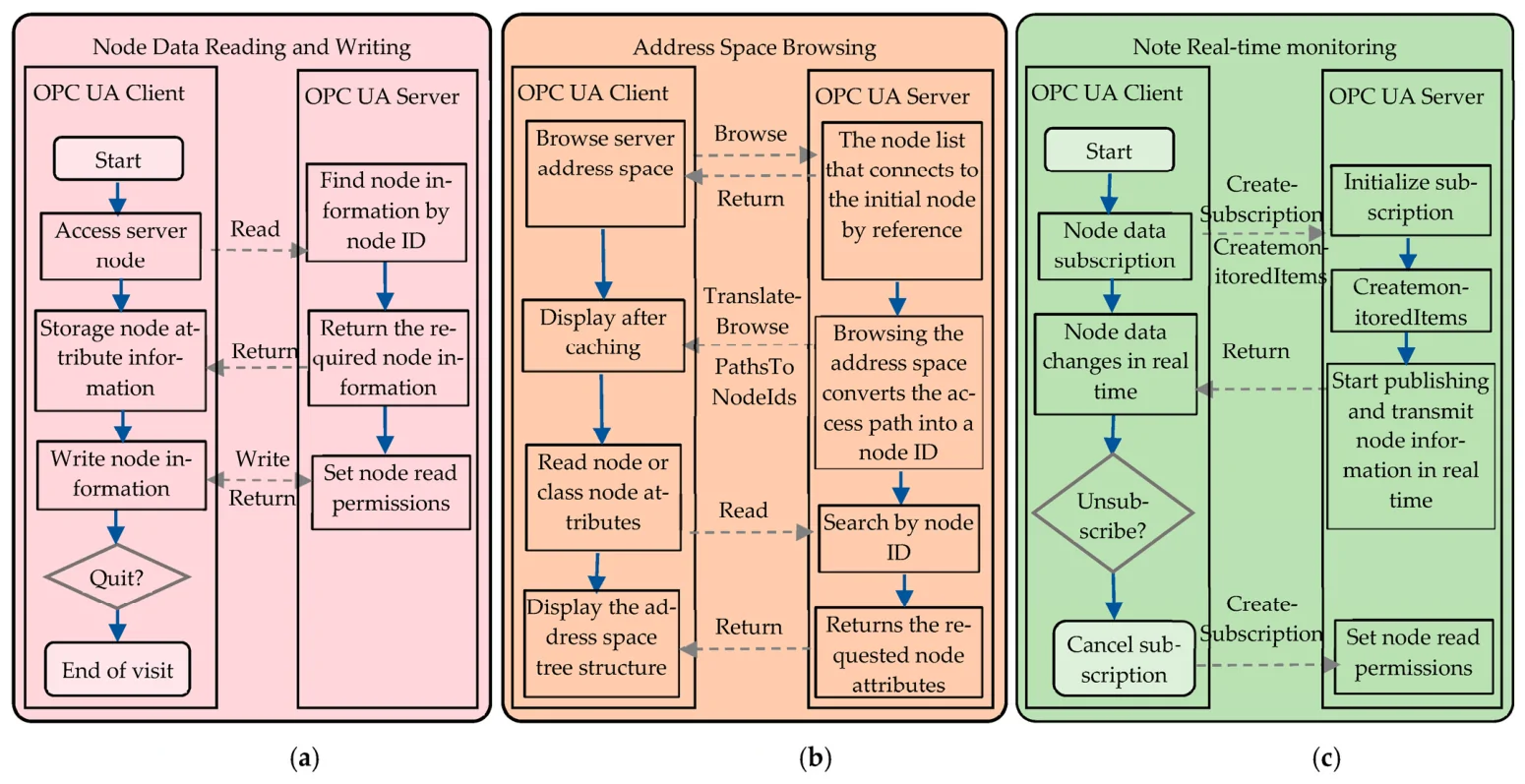
Schematic diagram of client-side-related components. (**a**) Node data read and write. (**b**) View server address. (**c**) Real-time monitoring of node data.

**Node Data Real-time Monitoring Component:** This component is primarily designed for dynamically changing data such as robot joint posture angles. It adopts the Subscription/MonitoredItem/Publish mechanism. After the client creates a subscription and adds monitored items, the server actively pushes data change notifications at a predefined interval, enabling real-time data updates and monitoring. The core principles of the above components are illustrated in Figure 11.

### 4.5. Cloud-Edge-End Collaborative Database Construction

#### 4.5.1. Database Table Design for Cloud–Edge–End Architecture

The database undertakes the data storage tasks for the grinding robot, covering four categories of data: robot properties, process perception information, process parameters, and user management information. Specifically, robot information describes the core attributes of the equipment (such as model, name, IP address, etc.). Process data mainly includes spark images captured by high-precision industrial cameras and acoustic data collected by sound sensors, supplemented by timestamps for recording. Belt grinding parameter data is used to store key process variables including abrasive grit size, belt width, belt material, speed, feed rate, grinding depth, and contact pressure. User data records information such as user ID, role, and permission level, which is deployed on the cloud to support cross-platform login and access control. All the above data are managed within a unified cloud–edge–end collaborative MySQL database system, with the complete information and primary key settings presented in Table 7.

#### 4.5.2. Design of Database Master–Slave Replication Under the Cloud–Edge–End Architecture

**Figure 12:**
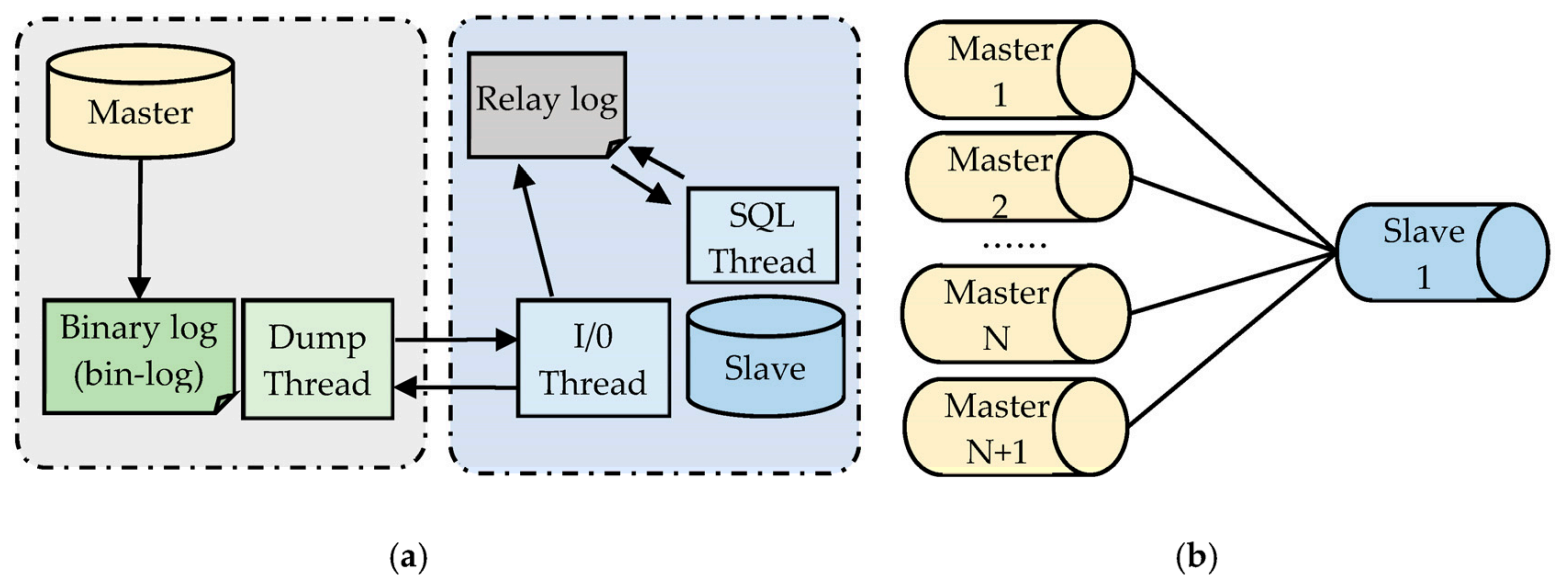
Master–Slave Replication Design Scheme for Cloud–Edge Database of Grinding Robot. (**a**) MySQL Master–Slave Replication Architecture Flowchart. (**b**) MySQL Multi-Master–One-Slave Schematic Diagram.

Building upon the communication-level data flow illustrated in Figure 9, this subsection further elaborates the database-level data flow across the three layers, as shown in Figure 12. This system adopts the MySQL 8.0.X master–slave replication architecture to achieve unified aggregation of data from multiple edge–node databases to the cloud node. The master–slave replication mechanism synchronizes data changes from the master database to the slave database in real time, thereby enabling centralized processing of multi-source data while allowing rapid failover to the slave database in the event of master failure to ensure service continuity. Additionally, this architecture facilitates horizontal scaling and read–write separation deployment. In terms of functional division, the master database handles update transactions such as insert, delete, and modify, while the slave database is dedicated solely to responding to query requests. MySQL supports three asynchronous replication modes, among which this system adopts the most widely used SQL-statement-based replication strategy, the workflow of which is illustrated in Figure 12a.

The master database writes data changes into the binary log (binlog). The slave database periodically polls the master’s binlog for updates; upon detection of new logs, it starts an I/O thread to fetch them. For each slave’s I/O thread, the master allocates a corresponding binlog dump thread responsible for log transmission. The slave writes the received logs into the relay log, after which the SQL thread reads, parses, and executes the SQL statements from the relay log, thereby ensuring data consistency between the master and the slave.

To address the requirement that multiple edge databases from grinding robots in this system need to be uniformly replicated to the cloud for centralized management, the system adopts a multi-master-to-one-slave deployment, i.e., a multi-source replication architecture, as shown in Figure 12b. During configuration, the master database must grant replication privileges to the slave database, and the initial configuration files of both the master and the slave need to be modified respectively (as listed in Table 8 and Table 9). The replication architecture takes effect after restarting the database services.

#### 4.5.3. Design of Read–Write Separation for Cloud–Edge–End Databases

**Figure 13:**
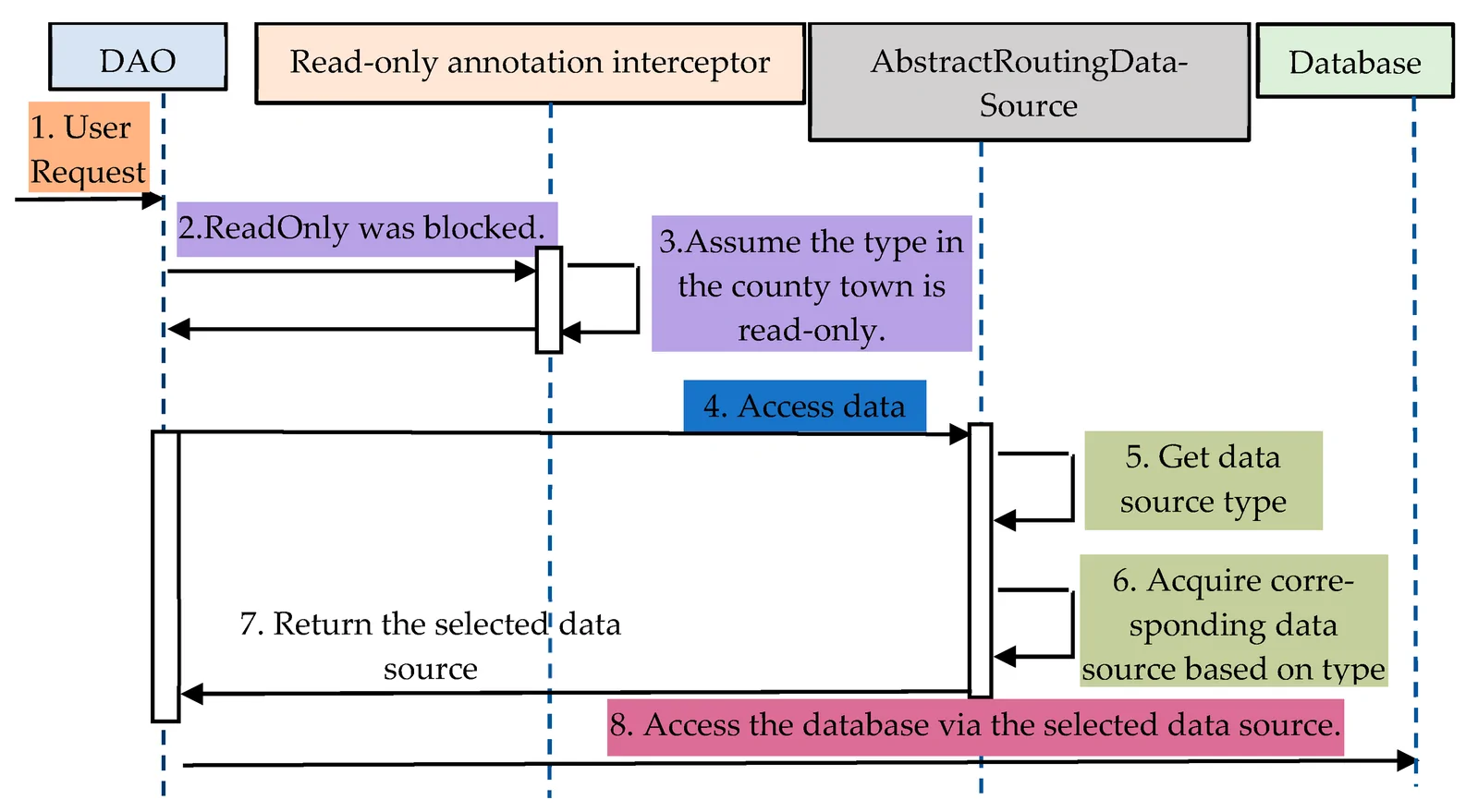
MySQL database read and write separation schematic.

Database operations that insert, delete, and modify data are collectively referred to as write operations, all of which alter data content. In concurrent scenarios, write operations may cause issues such as dirty reads, non-repeatable reads, and phantom reads. Although locking mechanisms can address these problems, they significantly degrade system performance. Since write operations occur far less frequently than read operations, excessive locking may unnecessarily block read operations, making read–write separation a critical strategy. Two main approaches exist for implementing read–write separation: code-level implementation, which results in tight coupling and high maintenance complexity, and proxy-based implementation, which offers sufficient decoupling but incurs higher costs. For the purpose of feasibility validation, this paper adopts the code-level approach, the principle of which—based on the SpringBoot environment—is illustrated in Figure 13.

It can be observed that the specific implementation process of MySQL database read–write separation is as follows. First, the connection information of the master and slave databases is stored in a HashMap in the form of key–value pairs, where the key represents the data source type (read/write), and when multiple slave databases are supported, they are distinguished by sequence numbers. Second, a custom “@ReadOnly” annotation is defined to mark methods that perform only read operations; before the method is executed, the current thread type is set to “read”, and the type setting is cleared after execution. Finally, the “AbstractRoutingDataSource” class provided by the SpringBoot framework is utilized to dynamically route and return the corresponding data source from the HashMap based on the current thread type, thereby ensuring that read operations access the slave database while write operations access the master database.

## 5. Digital Twin Monitoring Platform for Grinding Robot Grinding Process

### 5.1. Overall Scheme Design of the Platform

The data acquisition and monitoring client developed in this study is required not only to integrate the digital twin model of the grinding robot and the cloud–edge–end-based multi-source heterogeneous data transmission system but also to incorporate key functionalities such as a message broker server and real-time data curve monitoring in response to practical industrial application requirements. In doing so, the client achieves unified data management and real-time posture monitoring for the grinding robot, while demonstrating favorable scalability and adaptability to industrial field environments.

To achieve the above objectives, the client is required to satisfy the following four core functional requirements:

(1) **Client interactive interface:** Optimize user experience by clarifying the interface layout and control logic, reducing operational errors, and improving readability and usability.
(2) **Message broker server:** Based on the MQTT protocol, the client connection is maintained only during robot operation and disconnected during non-operation periods, thereby improving network resource utilization.
(3) **OPC UA communication:** Integrate the OPC UA client module with the robot monitoring platform to enable data stream transmission through reserved interfaces.
(4) **Machining data management and extraction:** Integrate with the cloud database to extract, store, and process spark images and acoustic data generated during the grinding operation.

**Figure 14:**
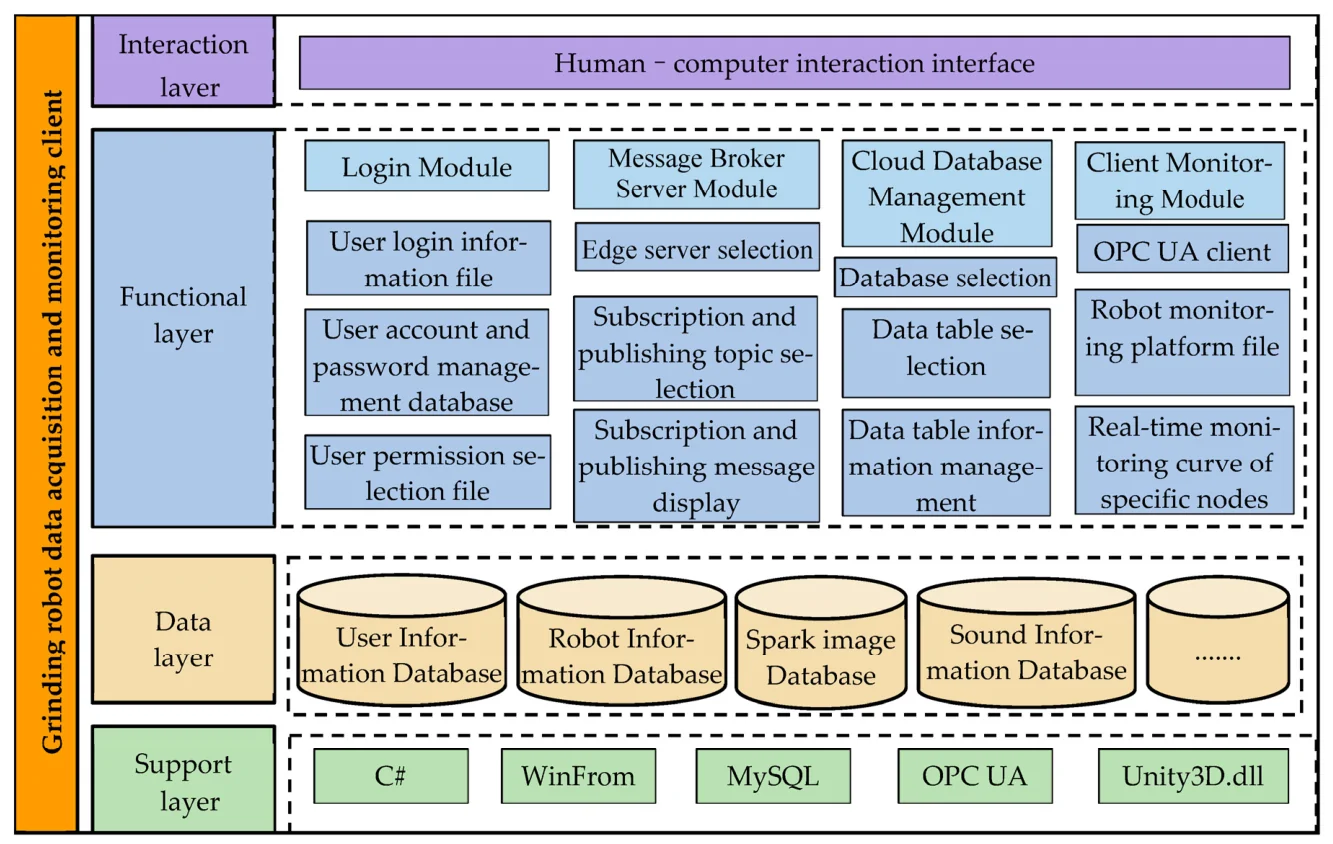
Overall framework of the polishing robot monitoring platform.

Based on the above system requirements analysis, a layered architecture is adopted for constructing the client. Specifically, the system is divided into four layers from top to bottom, namely the presentation layer, the functional execution layer, the data management layer, and the technical support layer. These layers work in coordination with one another to collectively support the stable operation of the entire system. The specific architecture of the client is illustrated in Figure 14.

As shown in Figure 14, the platform consists of four layers from top to bottom: the presentation layer, the functional layer, the data layer, and the support layer. Each layer has well-defined responsibilities, working collaboratively to ensure the efficient realization of system functions.

(1) **Presentation layer:** This layer is responsible for receiving user operation commands and forwarding them to the system core, while simultaneously returning response results to the user interface, thereby enabling bidirectional information exchange between the system and the user, and ensuring the immediacy and accuracy of operations.
(2) **Functional layer:** As the core business layer of the system, this layer integrates four core modules: login management, message broker service, cloud database management, and OPC UA client monitoring. Specifically, the cloud database management module undertakes the extraction and management of machining process data from the edge side; the OPC UA client monitoring module embeds the client into the monitoring platform and transmits the acquired data in real time in the form of data streams, thereby enabling continuous monitoring of the grinding robot’s operational status.
(3) **Data layer:** This layer is built upon a MySQL relational database and manages multiple data resources, including the user information database, robot information database, spark image information database, and acoustic information database, providing data support for the functional layer. Among these, the spark image and acoustic data are of significant reference value for evaluating the material removal rate and assessing the belt grinding status.
(4) **Support layer:** This layer provides the foundational technical environment for system development, encompassing core technology components such as the C# programming language, the WinForm graphical user interface framework, the MySQL database engine, and the OPC UA communication protocol stack, thereby offering a solid guarantee for the efficient development and stable operation of the client.

### 5.2. Development of the Grinding Robot Monitoring Interface

**Figure 15:**
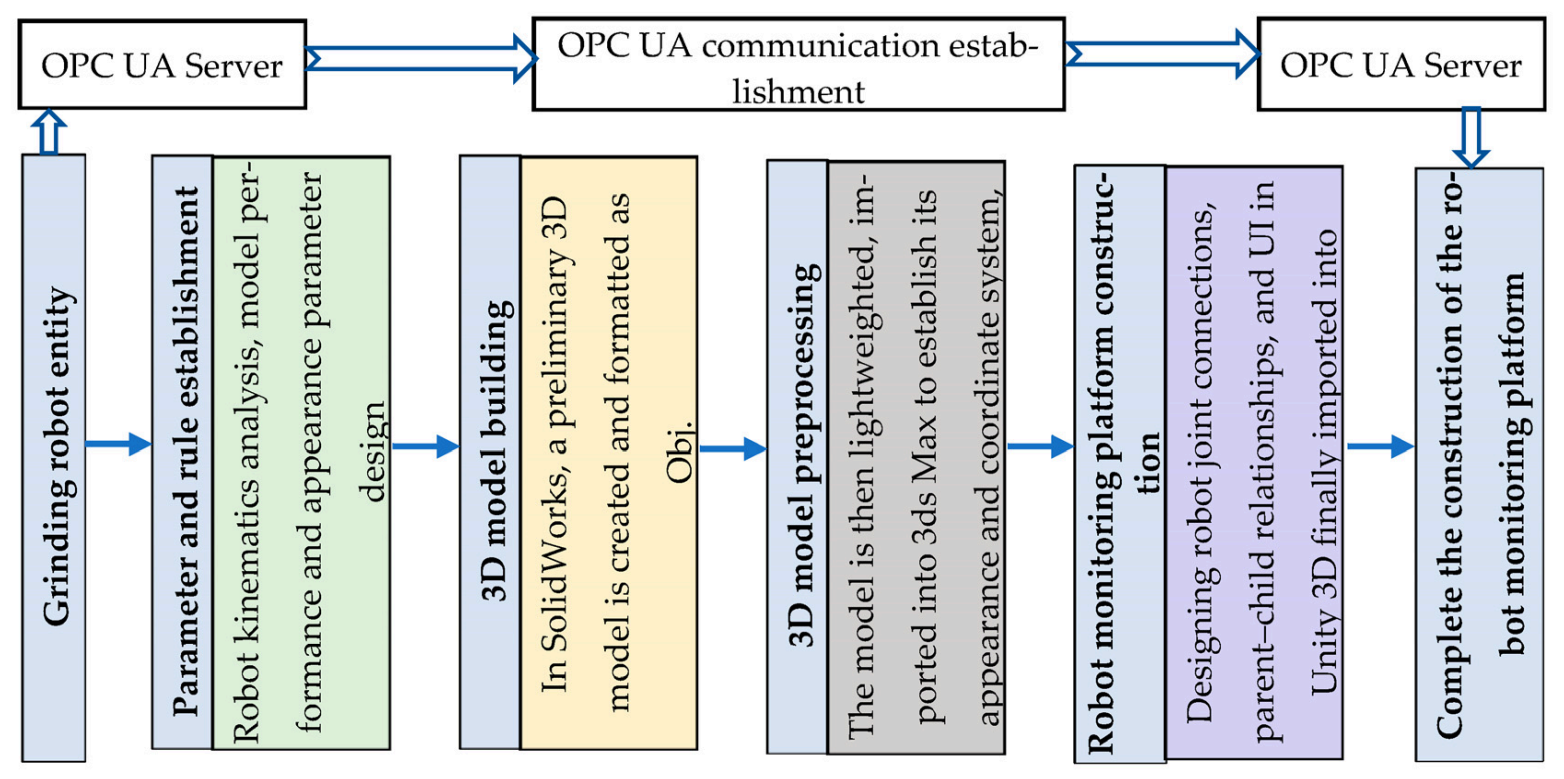
Construction scheme of the polishing robot monitoring platform.

The construction scheme of the grinding robot monitoring platform is shown in Figure 15, with its core functionality being the real-time posture display of the physical grinding robot within the monitoring platform. The construction process is as follows: First, an improved D-H coordinate system is established based on the motion rules of the physical robot, and both forward and inverse kinematics solutions are derived. Second, a preliminary 3D model is created according to the physical parameters of the robot; to enhance the operational fluency of the platform, lightweight processing is applied to certain model components to reduce computational resource consumption. The model is then imported into 3ds Max for detailed refinement, material adjustment, and establishment of the motion coordinate system, ensuring consistency between the virtual model and the physical robot in terms of motion. Finally, the processed model is imported into the Unity 3D engine, where joint parent–child hierarchy establishment, UI design (including the virtual robot display area, joint posture monitoring panel, warning panel, and communication connection panel), and C# motion script development are sequentially completed.

In terms of data interaction, the OPC UA client established in Section 4 is employed to acquire real-time joint posture angles from the physical grinding robot and transmit them to the monitoring platform in the form of data streams, thereby ensuring that the platform accurately and promptly obtains the actual operational status of the robot. Specifically, the joint posture monitoring panel displays the local Euler angles of each joint in real time via text components; the warning panel compares the received OPC UA data with the predefined workspace to determine whether the current operational status is within safe limits; and the communication connection panel enables real-time communication with the OPC UA client through button components and C# scripts, driving the virtual robot to dynamically map the movements of the physical robot.

This module integrates OPC UA communication technology with the grinding robot monitoring platform. By utilizing the OPC UA client to acquire and record joint posture angle data in real time and transmitting these data to the corresponding joints of the virtual robot, the module achieves real-time monitoring of the overall posture. Additionally, a node real-time curve monitoring function is developed, which intuitively displays the angular variation trends of subscribed joint nodes through two-dimensional line charts.

**Figure 16:**
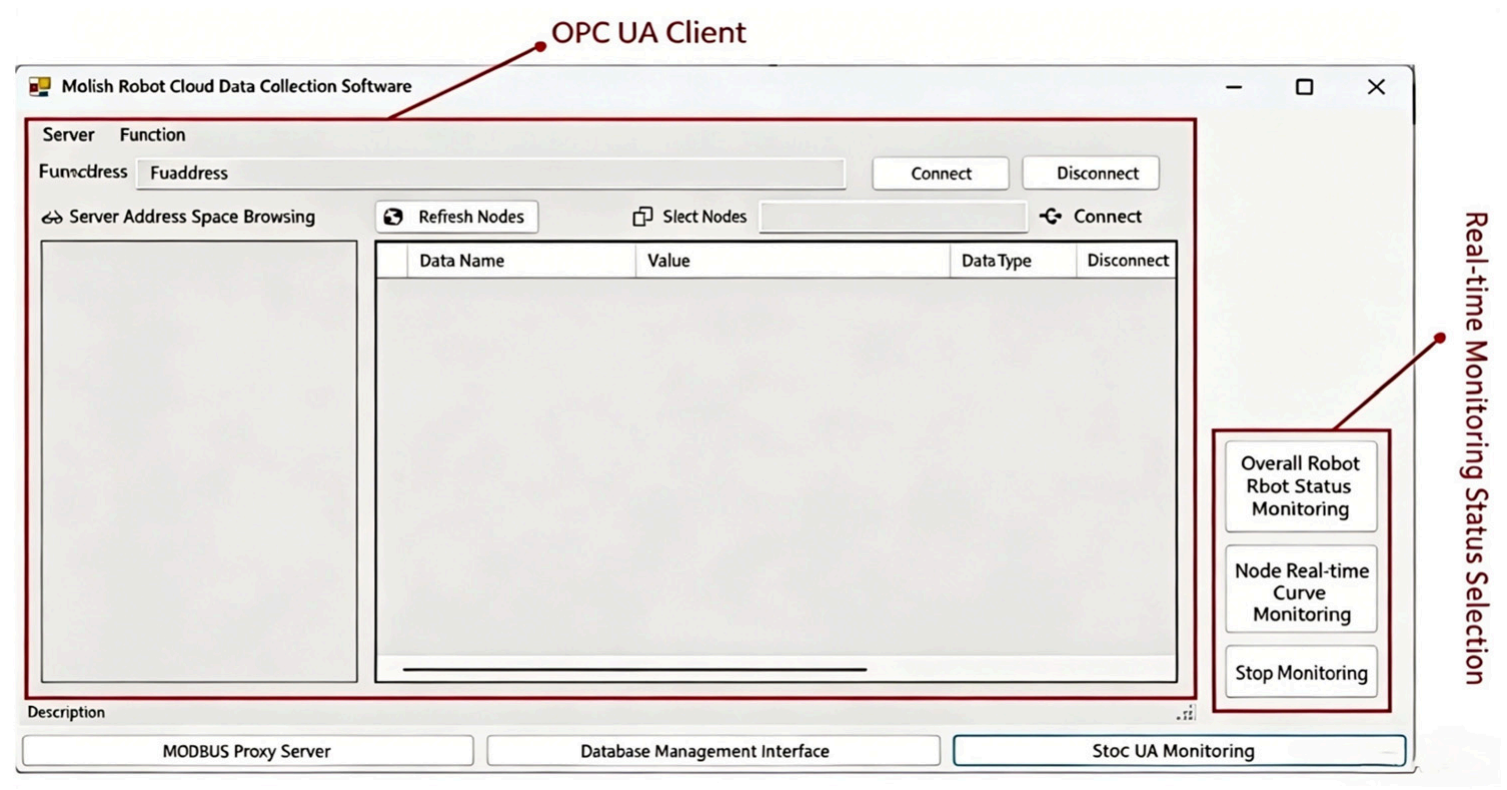
Real-time monitoring module interface.

The module interface is shown in Figure 16 and mainly consists of two parts: the OPC UA client area, which integrates all the functionalities of the aforementioned client to ensure stable server connections and efficient data transmission; and the real-time monitoring status selection area, which is enabled after the client successfully connects and reads node data, allowing users to choose between overall posture monitoring and single-node trend monitoring based on their requirements.

### 5.3. Real-Time Process Monitoring Platform for Grinding Robots

#### 5.3.1. OPC UA Client Login and Node Information Transmission Test

**Figure 17:**
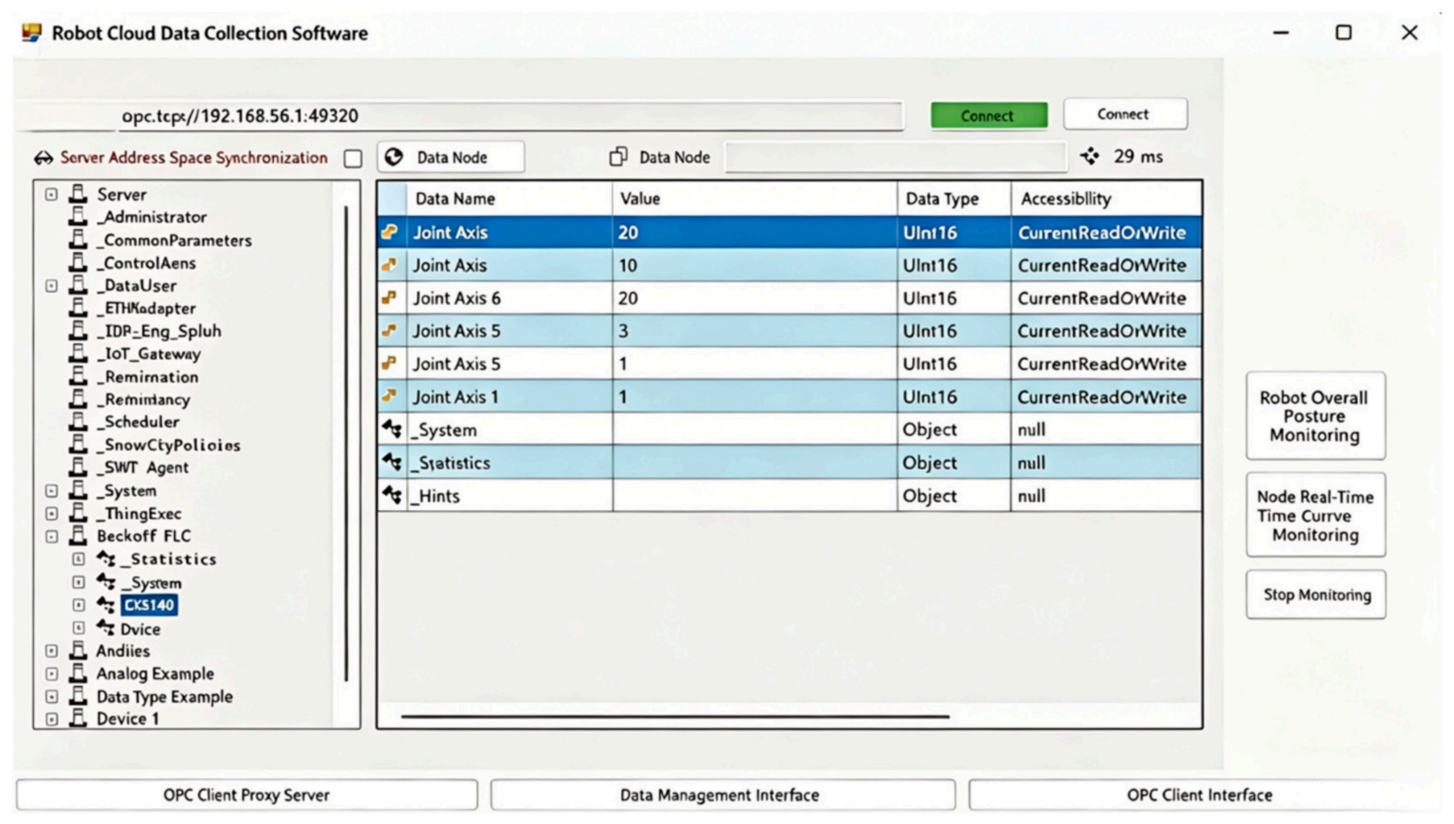
OPC UA client login and node information transfer test.

The following tests are first conducted on the functionality of the OPC UA client module in this subsystem, with a focus on verifying client connection establishment and real-time node data transmission. The OPC UA server, which has been preconfigured using KEPServerEX (TwinCAT 3Runtime) industrial software, is started. The server address “opc.tcp://192.168.56.1:49320” is entered in the server address field, and the connection button is clicked, with anonymous login selected according to the security policy. Subsequently, the CX5140 node of the Beckhoff PLC is selected in the address space browsing component (as shown in Figure 17). The node data information reading component then displays the successfully retrieved robot joint node data from the OPC UA server, indicating that the client has successfully connected to the server and completed normal login.

#### 5.3.2. Real-Time Joint Monitoring Function Test

Once the OPC UA client has completed connection establishment and node information operations, the real-time monitoring mode can be selected. The development of the node real-time curve monitoring function is first briefly introduced. In the program implementation, a custom photo class is defined, and the chart control is embedded into the form and instantiated as a component of the photo class. On this basis, the chart series attributes (Series) are configured, with the chart type set to line chart (Line), the X-axis set to the current system time, and the Y-axis set to represent the robot joint angle values. Additional attributes such as data point size and color are also configured. To enable dynamic data updates, a timer control is introduced with an interval of 1 s, serving to periodically refresh the Y-axis data of the line chart, ensuring that changes in the subscribed node data are reflected on the chart with low latency.

**Figure 18:**
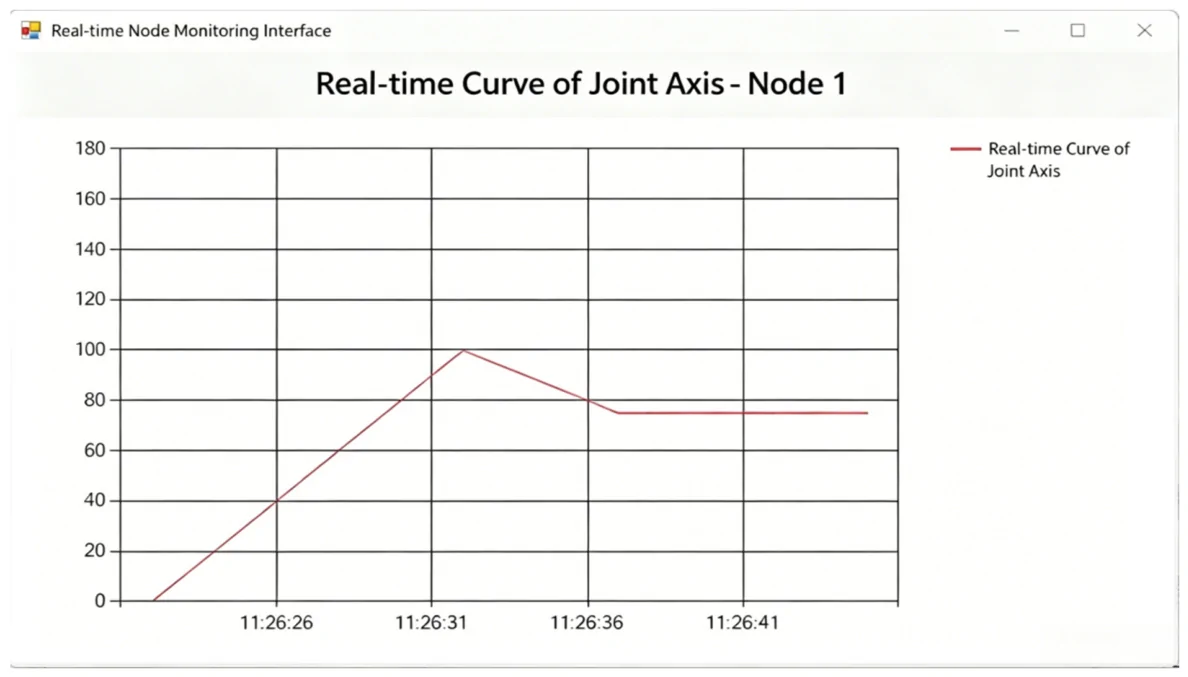
Real-time node curve monitoring test.

Taking the Joint Axis 1 node subscribed by the client as an example, the real-time curve monitoring function is tested, with the results shown in Figure 18. The test results demonstrate that this function can successfully display the variation trends of Joint Axis 1, meeting the requirements for real-time curve chart visualization.

#### 5.3.3. Testing of the Overall Robot Posture Monitoring Function

**Figure 19:**
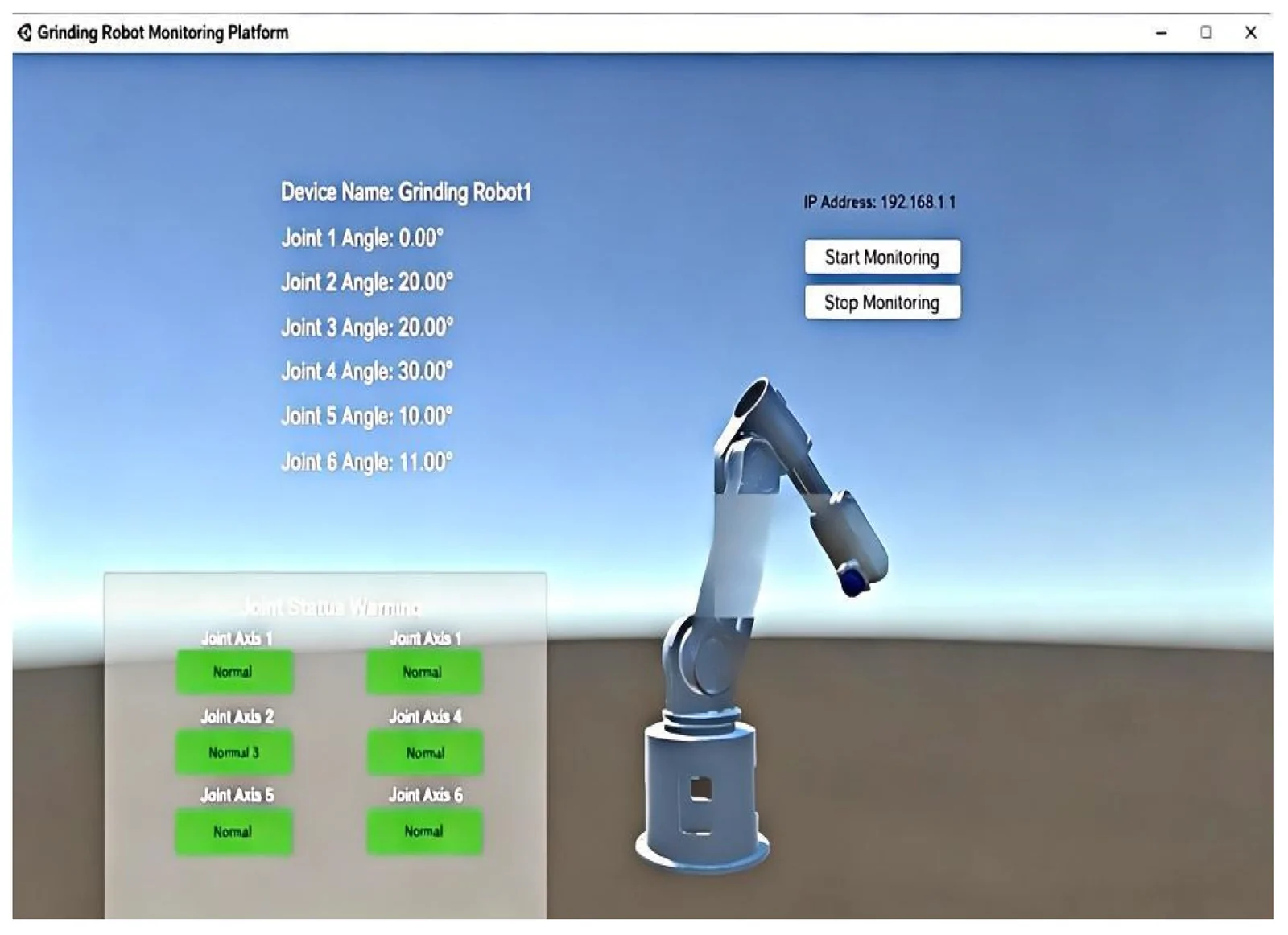
Robot overall attitude monitoring test.

The testing of the overall robot posture monitoring function can only be enabled after the OPC UA client has successfully read the data. The test results are shown in Figure 19.

The test results indicate that the overall robot posture monitoring function can successfully read the joint angle data transmitted by the OPC UA client and display it correctly on the monitoring interface. Meanwhile, the posture deviation between the virtual robot and the physical robot remains minimal. The above test results demonstrate that this module can effectively acquire the joint angle information of the physical robot and realize real-time monitoring through multiple approaches, thereby meeting the functional requirements of the system.

## 6. Conclusions

Taking a self-developed abrasive belt grinding robot as a case study, this study designs and develops a cloud–edge–end-based digital twin framework for grinding robots. The main conclusions are as follows:

(1) A digital twin framework model of the abrasive belt grinding robot was established. A virtual robot was first constructed, and the QEM algorithm was introduced to perform lightweight processing on the 3D robot model. The robot was modeled using the improved D-H method, based on which the forward and inverse kinematics solutions were derived. A grinding robot monitoring platform was developed using the Unity 3D virtual engine, enabling visualization of the robot’s motion status and posture data monitoring.
(2) A multi-source heterogeneous data acquisition and transmission framework for the grinding robot was established. An OPC UA server was built using KEPServerEX industrial automation software, with its internal Advanced Tags functionality enabling data exchange between different devices. The OPC UA client functionality was developed using the SDK provided by OPC UA.
(3) A cloud–edge–end database was established using MySQL database software. Various database tables were designed in detail according to the requirements of different data types. Subsequently, the multi-master-to-one-slave (multi-source replication) approach was adopted to aggregate data from edge-side databases to the cloud, and a read–write separation strategy was implemented on the database to improve operational efficiency.
(4) A digital twin monitoring platform for the grinding robot was developed using the WinForm framework. By integrating the data acquisition and transmission architecture, the cloud–edge–end database, and the digital model of the grinding robot, a digital twin monitoring platform for the abrasive belt grinding process was established. Finally, functional tests were conducted on selected modules, and the results demonstrate that the client meets the operational requirements. This digital twin framework for the grinding robot grinding process can be applied to virtual monitoring or real-time 3D visual representation of the robot.

However, this paper only develops the fundamental framework of the digital twin for the blade grinding robot, and certain limitations exist in this work. Developing a comprehensive digital twin that encompasses all aspects of the blade abrasive belt grinding robot process is both costly and time-consuming. Future work will build upon the proposed digital twin framework through the development of force models and tool wear prediction. Artificial intelligence algorithms will be implemented to predict system behavior and optimize process efficiency. In the future, we plan to introduce the federated learning framework into the proposed digital twin monitoring system, using local data from grinding robots distributed across multiple factories or workshops to jointly train tool wear prediction and process parameter optimization models. This will improve the generalization ability and robustness of the global model while protecting the data privacy of all participants.

## Figures and Tables

**Table 1:** Technical parameters of Beckoff CX5140-0050 industrial computer.

Parameter	Specification
Dimension	142 mm × 100 mm × 91 mm
Processor	Intel Atom E3845, 1.91 GHZ
Memory	4 GB DDR3 RAM
Operating System	Microsoft Windows Embedded Standard 7 P
Communication Bus	EtherCAT
Interfaces	2 × Ethernet RJ45 interfaces, 1 × DVI-I display interface, 4 × USB2.0 interfaces
Power Supply Unit	1 × 24 V controller and terminal power supply unit

**Table 2:** Number of triangular surfaces before and after model simplification.

Model Name	Joint Axis 1	Joint Axis 2	Joint Axis 3	Joint Axis 4	Joint Axis 5
Number of triangular facets before weight reduction	11,942	25,080	11,338	10,288	7796
Number of triangular facets after weight reduction	1264	2483	1112	978	757

**Table 3:** Modified meaning of D-H method parameters.

Symbol Name	Describe
$a_{i-1}$	The distance from $Z_{i-1}$ to $Z_{i}$ along the $X_{i-1}$ direction (the length of the common perpendicular between the two coordinate systems).
$\alpha_{i-1}$	Starting from the $X_{i-1}$ direction, the selected rotation angle from $Z_{i-1}$ to $Z_{i}.$
$d_{i}$	Along the $Z_{i}$ direction, the distance from $X_{i-1}$ to $X_{i}$ (the distance between the common perpendiculars of the two coordinate systems).
$\theta_{i}$	Rotation angle from $X_{i-1}$ to $X_{i}$ starting from the $Z_{i}$ direction.

**Table 4:** Parameter table of robot modified D-H method.

Joint ( i )	$a_{i-1}\;(mm)$	$\alpha_{i-1}\;({}^{\circ})$	$d_{i}\;(mm)$	$\theta_{i}\;({}^{\circ})$	Variable
1	0	0	618	0	$\theta_{1}$
2	700	90	0	90	$\theta_{2}$
3	230	0	−46	0	$\theta_{3}$
4	0	90	792	0	$\theta_{4}$
5	0	−90	0	0	$\theta_{5}$
6	0	90	0	0	$\theta_{6}$
7	0	0	198	0	0

**Table 5:** The results of inverse kinematics are derived from the angles of each joint.

Joint Angle	Calculation Results
$\theta_{1}$	$A\tan2\left(p_{y},p_{x}\right)-A\tan2\left(\frac{d_{3}}{\rho },\pm \sqrt{1-{\left(\frac{d_{3}}{\rho }\right)}^{2}}\right)$
$\theta_{2}$	$A\tan2\left[\begin{array}{c} (a_{2}c_{3}+a_{3})(p_{z}-d_{1})+(d_{4}+a_{2}s_{3})(c_{1}p_{x}+s_{1}p_{y}),\\ -(d_{4}+a_{2}s_{3})(p_{z}+d_{1})+(a_{2}c_{3}+a_{3})(c_{1}p_{x}+s_{1}p_{y}) \end{array}\right]-\theta_{3}$
$\theta_{3}$	$A\tan2(\frac{k}{\rho },\pm \sqrt{1-{\left(\frac{k}{\rho }\right)}^{2}})-A\tan2(a_{3},d_{4})$
$\theta_{4}$	$\left\{\begin{array}{c} A\tan2\left(a_{x}s_{1}-a_{y}c_{1},a_{x}c_{1}c_{23}+a_{y}s_{1}c_{23}+a_{z}s_{23}\right)\\ \mathrm{Sin}\mathrm{gularity} \end{array}\right.\begin{array}{c} \theta_{5}\neq 0\\ \theta_{5}=0 \end{array}$
$\theta_{5}$	$A\tan2\left(s_{5},c_{5}\right)$
$\theta_{6}$	$A\tan2\left(s_{6},c_{6}\right)$

**Table 6:** OPC UA target variable property configuration.

OPC UA Property	Variable Properties	Describe
Data Access (DA)	{attribute ‘OPC.UA.DA’ := ‘0’}	After disabling an OPC UA variable, the variable becomes invisible in the UA namespace.
Data Access (DA)	{attribute ‘OPC.UA.DA’ := ‘1’}	After enabling an OPC UA variable, the variable becomes visible in the UA namespace.
Data Access (DA)	{attribute ‘OPC.UA.DA.Access’ := ‘x’}	Set the read/write access rights for the variable according to parameter “x”; 0 = no access, 1 = read-only, 2 = write-only, 3 = read/write (default is 3 if no tag is used).
Data Access (DA)	{attribute ‘OPC.UA.DA.Description’ := ‘x’}	Set the text for the OPC UA property”Description”.
Data Access (DA)	{attribute ‘OPC.UA.DA.StructuredType’ := ‘0’}	Disable the StructuredType of a structure or function block.
Data Access (DA)	{attribute ‘OPC.UA.DA.StructuredType’ := ‘1’}	Enable the StructuredType of a structure or function block.
Data Access (DA)	{attribute ‘OPC.UA.DA.Status’ := ‘quality’}	Manually define the StatusCode of a symbol in the UA namespace, where “quality” specifies which DINT subelement of the data is addressed.
Historical Visits (HA)	{attribute ‘OPC.UA.HA’ := ‘1’}	Enable the “History Access” feature for the variable.
Historical Visits (HA)	{attribute’OPC.UA.HA.Storage’ := ‘x’}	Define the storage location for history access according to parameter “x”: 1 = memory, 2 = file, 3 = SQL compact database, 4 = SQL Server database.
Historical Visits (HA)	{attribute’OPC.UA.HA.Sampling’ := ‘x’}	Define the sampling rate for saving variable values according to parameter “x”, in milliseconds (ms).
Historical Visits (HA)	{attribute ‘OPC.UA.HA.Buffer’ := ‘x’}	Define the maximum number of values to be retained in the data memory according to parameter “x”.

**Table 7:** Field definitions of core data tables in the cloud–edge–end database.

Information Type	Field Name	Type	Length	Nullable	Primary Key	Description
Robot Information	Name	varchar	20	No	Yes	Robot name
	Version	varchar	20	No	No	Robot model
	IP	varchar	20	No	No	Robot IP address
	Manufacturer	varchar	20	No	No	Manufacturer
	FactoryDate	varchar	20	Yes	No	Factory date
	Usedate	varchar	20	Yes	No	Commissioning date
	Type	varchar	10	No	No	Robot type
Spark Image Information	Number	int	2	No	Yes	Robot ID
	Date	varchar	30	Yes	No	Processing date
	Time	varchar	30	Yes	No	Acquisition time
	Image	longblob	—	Yes	No	Spark image
	Remarks	varchar	50	No	No	Remarks
Sound Data Information	Number	int	2	No	Yes	Robot ID
	Date	varchar	30	Yes	No	Processing date
	Sound	longblob	—	Yes	No	Sound data
	Remarks	varchar	50	No	No	Remarks
Belt Grinding Parameter Information	Number	int	2	No	Yes	Robot ID
	Mesh	varchar	20	Yes	No	Abrasive grain size
	Broadband	varchar	20	Yes	No	Belt width
	Materials	varchar	20	No	No	Belt material
	Velocity	varchar	20	Yes	No	Belt speed
	Feedrate	varchar	20	Yes	No	Feed rate
	Depth	varchar	20	Yes	No	Grinding depth
	Pressure	varchar	20	No	No	Grinding pressure
User Data Information	ID	int	10	No	Yes	User ID
	Username	varchar	20	Yes	No	Username
	Password	varchar	20	Yes	No	Password
	Level	int	10	Yes	No	User level
	Role	varchar	20	Yes	No	User role
	Permission	text	—	Yes	No	User permission

**Table 8:** Master database configuration.

Configuration Item	Reference Value	Description
Server-id	1	Unique ID of the master database
Log-bin	Master-bin	Binary log filename and its storage path
Binlog-do-db	Slave1	Name of the database to be logged and replicated

**Table 9:** Slave database configuration.

Configuration Item	Reference Value	Description
Server-id	2	Unique ID of the slave database
Relay-log	slave-relay	Relay log filename and its storage path

## Data Availability

The original contributions presented in this study are included in the article. Further inquiries can be directed to the corresponding author.
